# Hypothermia Prevents Cardiac Dysfunction during Acute Ischemia Reperfusion by Maintaining Mitochondrial Bioenergetics and by Promoting Hexokinase II Binding to Mitochondria

**DOI:** 10.1155/2022/4476448

**Published:** 2022-07-13

**Authors:** Jie Sun, Jyotsna Mishra, Meiying Yang, David F. Stowe, James S. Heisner, Jianzhong An, Wai-Meng Kwok, Amadou K. S. Camara

**Affiliations:** ^1^Department of Anesthesiology, Research Division, Medical College of Wisconsin, USA; ^2^Institute of Clinical Medicine Research, Department of Gastroenterology and Hepatology, Suzhou Science and Technology Town Hospital, Suzhou, Jiangsu, China; ^3^Department of Physiology, Medical College of Wisconsin, Milwaukee, WI, USA; ^4^Department of Biomedical Engineering, Medical College of Wisconsin and Marquette University, Milwaukee, WI, USA; ^5^Cardiovascular Center, Medical College of Wisconsin, Milwaukee, WI, USA; ^6^Department of Pharmacology and Toxicology, Medical College of Wisconsin, Milwaukee, WI, USA; ^7^Cancer Center, Medical College of Wisconsin, Milwaukee, WI, USA

## Abstract

**Background:**

Hypothermia (H), cardioplegia (CP), and both combined (HCP) are known to be protective against myocardial ischemia reperfusion (IR) injury. Mitochondria have molecular signaling mechanisms that are associated with both cell survival and cell death. In this study, we investigated the dynamic changes in proapoptotic and prosurvival signaling pathways mediating H, CP, or HCP-induced protection of mitochondrial function after acute myocardial IR injury.

**Methods:**

Rats were divided into five groups. Each group consists of 3 subgroups based on a specific reperfusion time (5, 20, or 60 min) after a 25-min global ischemia. The time control (TC) groups were not subjected to IR but were perfused with 37 °C Krebs-Ringer's (KR) buffer, containing 4.5 mM K^+^, in a specific perfusion protocol that corresponded with the duration of each IR protocol. The IR group (control) was perfused for 20 min with KR, followed by 25-min global ischemia, and then KR reperfusion for 5, 20, or 60 min. The treatment groups were exposed to 17 °C H, 37 °C CP (16 mM K^+^), or HCP (17 °C + CP) for 5 min before ischemia and for 2 min on reperfusion before switching to 37 °C KR perfusion for the remainder of each of the reperfusion times. Cardiac function and mitochondrial redox state (NADH/FAD) were monitored online in the *ex vivo* hearts before, during, and after ischemia. Mitochondria were isolated at the end of each specified reperfusion time, and changes in O_2_ consumption, membrane potential (*ΔΨ*_m_), and Ca^2+^ retention capacity (CRC) were assessed using complex I and complex II substrates. In another set of hearts, mitochondrial and cytosolic fractions were isolated after a specified reperfusion time to conduct western blot assays to determine hexokinase II (HKII) and Bax binding/translocation to mitochondria, cytosolic pAkt levels, and cytochrome *c* (Cyto-*c*) release into the cytosol.

**Results:**

H and HCP were more protective of mitochondrial integrity and, concomitantly, cardiac function than CP alone; H and HCP improved post-ischemic cardiac function by (1) maintaining mitochondrial bioenergetics, (2) maintaining HKII binding to mitochondria with an increase in pAkt levels, (3) increasing CRC, and (4) decreasing Cyto-*c* release during reperfusion. Bax translocation/binding to mitochondria was unaffected by any treatment, regardless of cardiac functional recovery.

**Conclusions:**

Hypothermia preserved mitochondrial function and cardiac function, in part, by maintaining mitochondrial bioenergetics, by retaining HKII binding to mitochondria via upstream pAkt, and by reducing Cyto-*c* release independently of Bax binding to mitochondria.

## 1. Introduction

Cardiac ischemia reperfusion (IR) injury results when a disease-induced blockage of blood flow in a major coronary vessel is relieved by reperfusion. IR injury can also occur during coronary angioplasty, cardiac valve replacement, or coronary bypass grafting [1]. Following cardiac ischemia, the most effective strategy for mitigating injury and limiting infarction is early restoration (reperfusion) of coronary blood flow to the ischemic myocardium [2–5]. However, this approach, while essential, is often associated with functional and structural damage to the myocardium because of the reperfusion damage that occurs the longer the ischemia [2, 6, 7]. Hence, timely reperfusion during acute ischemia is crucial for salvage of the ischemic myocardium. Unfortunately, reperfusion leads to the activation of many signaling pathways that contribute independently to both apoptotic and necrotic tissue injury and thus decreases the amount of viable myocardium. Reperfusion mediated mitochondrial damage is usually characterized by increased reactive oxygen species (ROS) production, cytosolic-induced mitochondrial Ca^2+^ overload, a decreased NADH/FAD redox ratio, and formation and opening of the deleterious mitochondria permeability transition pore (mPTP) [7].

Mitochondria are key regulators of cellular metabolism in the heart; but their dysfunction during cardiac IR injury can lead to cardiac dysfunction and myocyte demise [8]. The importance of mitochondria as both targets and mediators of IR injury is now well recognized. Pharmacological and nonpharmacological interventions to mitigate mitochondrial damage during IR have proven to be somewhat beneficial in rescuing the myocardium from IR injury, particularly if the intervention is applied before or during the ischemia. For example, we have reported that amobarbital, a drug that attenuates respiration at complex I [9], and ranolazine, a clinical antianginal drug that reduces cell and mitochondrial Ca^2+^ overload, and indirectly reduces ROS production with IR injury [7], are both cardioprotective in part by preserving mitochondrial function. We also reported previously that nonpharmacological interventions, like hypothermia (H), cardioplegia (CP), and HCP, provide varying degrees of protection against IR injury by attenuating excess mitochondrial and cytosolic Ca^2+^ and by reducing deleterious mitochondrial ROS production [10–13]. The mitochondrial mechanisms underlying protection against acute IR injury by H and CP have not been investigated during early and late reperfusion. A better understanding of the mitochondrial mechanisms that provide protection by H, CP, or HCP may enhance the organelle's therapeutic potential and the timing for improving cardioprotection after IR injury.

Clinically, both H and CP are utilized as effective interventions to protect the heart against ischemic injury during cardiac surgery. CP provides a motionless field by arresting the heart and H reduces cardiac metabolism even while the heart is not contracting; switching from cold CP perfusate to warm blood reperfusion allows for effective post-ischemic myocardial resuscitation [14]. Even though the effect of cooling on reducing overall metabolism is well known, the mitochondrial mechanisms by which H attenuates IR injury remain to be elucidated. Hypothermic-induced protection during reperfusion may be linked to a myriad of effects, including diminished ROS production and decreased cytosolic and mitochondrial Ca^2+^ overload [15], lower lactate levels from anaerobic metabolism, decreased cellular acidosis [4], and decreased energy utilization during ischemia that preserves essential mechanisms for rapid regeneration of ATP on reperfusion. Hypothermia has also been reported to modulate cell survival signaling pathways such as the Akt pathway [16]. CP generally entails the use of a hyperkalemic perfusate to depolarize all heart cells in synchrony, which provides diastolic cessation of all electromechanical activity and reduced myocardial O_2_ consumption.

In a previous report, we showed that normothermic CP decreased ROS generation during IR and better maintained a reduced mitochondrial redox state (NADH and FADH_2_) [11]. Hypothermic CP (HCP) [10] is widely used for coronary artery bypass grafting because it not only abruptly stops the heart but also reduces the cardiac energy demand. The central roles of H, CP, and HCP on altering mitochondrial function as both a target and initiator of signaling cascades that lead to cardiac protection during IR injury are not well described. A better understanding of changes in signaling pathways induced by H, CP, and HCP that mediate mitochondrial adaptation to IR injury could lead to new therapeutic approaches that specifically target mitochondria to protect the heart from IR injury.

We hypothesized that perfusion of CP, H, or HCP just before and after ischemia attenuates myocardial IR injury by initiating key antiapoptotic proteins/signaling molecules that are important in promoting mitochondrial-mediated cell survival on reperfusion. In addition, we postulated that activation of these signaling molecules is dependent on the duration of the reperfusion time at which protection is elicited. In planning our approach, we sought to define the chronological order of signaling events that promote or mitigate damage to mitochondria and confer cardiac dysfunction or protection, respectively. Factors that we looked at were as follows: (1) the comparative protective effects of H, CP, and HCP on hexokinase II (HKII) binding to mitochondria over time during reperfusion mediated by an Akt mechanism; (2) the role of the proapoptotic protein Bax translocation/binding to mitochondria; and (3) cytochrome *c* (Cyto-*c*) release, a marker of apoptosis. We found that H, rather than CP, is the most important intervention in maintaining HKII binding to mitochondria likely via a pAkt mechanism [16] and in attenuating Cyto-*c* release. Bax translocation/binding to mitochondria was not affected. These mitochondrial events appear to be important contributing factors to the protection afforded by hypothermia.

## 2. Materials and Methods

### 2.1. Isolated Heart Preparation and Measurements

All experiments conformed to the NIH “Guide for the Care and Use of Laboratory Animals” (NIH Publication N0. 85-23, Revised in 1996) for humane care and was approved by the Institutional Animal Care Committee of the Medical College of Wisconsin (MCW). Male Sprague-Dawley rats (weight: 250 to 300 g; *n* = 81), aged 10-12 weeks, were housed at the MCW animal facility in rooms set at 25 °C with 60% humidity under a 12-h light-dark cycle. Rats were allowed access to food and water *ad libitum*.

The methods described here have been detailed in our previous studies [7, 10, 12, 13, 17–22]. In brief, rats were injected intraperitoneally with a combination of ketamine (50 mg/kg) for anesthesia, and 1000 units heparin to prevent blood coagulation. Rats were decapitated only when unresponsive to a noxious stimulus to the hind limb. Following decapitation and thoracotomy, the heart was rapidly excised, and the aorta was cannulated and immediately perfused retrograde, with chilled oxygenated Krebs Ringer's (KR) buffer. After proper placement of the aortic cannula, the heart was immediately transferred to a Langendorff apparatus for further perfusion under a constant pressure with a KR solution (37 °C) that contained in mM, 138 Na^+^, 4.5 K^+^, 1.2 Mg^2+^, 2.5 Ca^2+^, 134 Cl^−^, 15 HCO^3-^, 1.2 H_2_PO^4-^, 11.5 glucose, 2 pyruvate, 16 mannitol, 0.1 probenecid, 0.05 EDTA, and 5 U/L insulin, and maintained at pH 7.4 and gassed with a 3% CO_2_+97% O_2_.

A physiological saline-filled latex balloon attached to a transducer was inserted into the left ventricle, via the left atrium, to measure left ventricular systolic and diastolic pressures (LVP). At the start of each experiment, the balloon volume was adjusted to set the diastolic LVP to 0 mmHg so that any subsequent changes in diastolic LVP represents an index of left ventricular contracture attributable to IR damage. The systolic and diastolic LVPs were monitored continuously online. Developed LVP (dLVP) was derived from the difference between systolic LVP and diastolic LVP. The rate of contractility (*d*LVP/*d*t_max_) and relaxation (*d*LVP/*d*t_min_) were derived from the systolic LVP. Coronary flow (CF) was measured by an ultrasonic flowmeter (model T106X; Transonic Systems, Ithaca, NY) placed directly into the aortic inflow line. Spontaneous heart rate (HR) was monitored with bipolar electrodes placed in the right atrial and ventricular free-walls. The rate pressure product (RPP), an index of cardiac work, was calculated as the product of the dLVP and HR (RPP = *d*LVP × HR) [23, 24]. Coronary inflow and outflow ionic conditions (Na^+^, K^+^, and Ca^2+^), pH, partial pressures of O_2_, and CO_2_ were measured offline with an intermittently self-calibrating analyzer system (Radiometer Copenhagen ABL 505; Copenhagen, Denmark) [12].

Indices of mitochondrial redox state, i.e., NADH and FAD derived from tissue autofluorescence signals [12, 13], were measured online by fluorescence spectrophotometry (Qm-8, Photon Technology Instrument; HORIBA Scientific, Piscataway, NJ) in a light-proofed Faraday cage. A trifurcated fiberoptic probe (3.8 mm^2^/bundle) was placed gently against the left ventricular free-wall, as described previously [12, 13, 25]. In the cardiomyocyte, the majority of the NADH is generated from mitochondria, with negligible amount emanating from glycolysis. FAD is primarily from mitochondria.

### 2.2. Experimental Groups and Protocols

**Figure 1:**
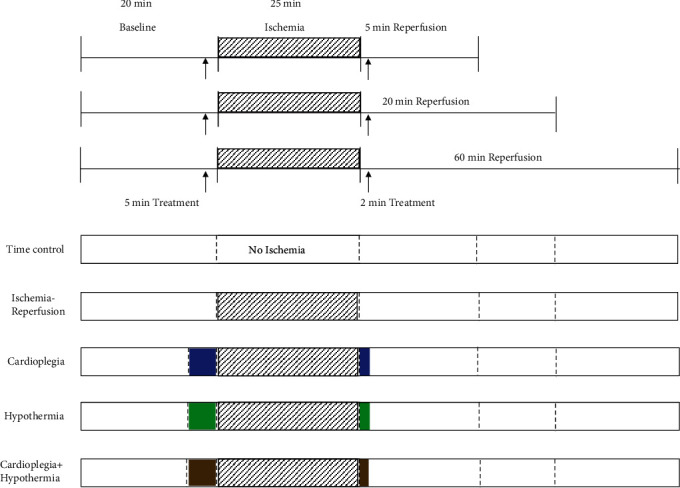
Timeline protocols for the time control (no ischemia), ischemia reperfusion (grey patterned box) ± cardioplegia (blue box), hypothermia (green box), or cardioplegia + hypothermia (brown box) treatment groups with 5, 20, or 60 min reperfusion after 25 min of global ischemia.

Rats were divided into five groups. Each group has three subgroups based on the reperfusion times of 5, 20, or 60 min after the global ischemia. The five groups were as follows: time control (TC), IR (normothermic ischemia, no treatment), hypothermia (H), cardioplegia (CP), and H + CP. In the TC groups, hearts were perfused with KR for the duration of an IR protocol (Figure 1) without ischemia (n = 3 in each subgroup). The relative constancy of the functional variables during the TC perfusions validated the functional stability of our *ex vivo* perfused heart model. In the IR group (no treatment; control), after 20 min KR perfusion for stabilization, the hearts underwent 25-min global ischemia, followed by warm (37 °C) KR (4.5 mM K^+^) reperfusion for 5, 20, or 60 min (*n* = 6 hearts/subgroup). In the H group (*n* = 6 hearts/subgroup), the hearts were subjected to similar KR perfusion for stabilization followed by 5 min cold KR (17 °C) perfusion before initiating cold global ischemia for 25 min. In the CP group (*n* = 6 hearts/subgroup), the hearts were perfused with high [K^+^] KR solution at 37 °C (CP; KR with 16 mM K^+^) for 5 min before 25-min global warm ischemia. In the HCP (17 °C) group (N= 6 hearts/subgroup), the hearts were perfused with HCP for 5 min before the 25-min HCP global ischemia. During reperfusion, hearts were perfused with their respective treatments for 2 min before switching back to normal warm (37 °C) KR perfusion for the remaining 5, 20, or 60-min reperfusion periods. At the end of each specified reperfusion time (5, 20, or 60 min), the hearts were immediately removed from the perfusion apparatus, and mitochondria were isolated for assessment of mitochondrial bioenergetics, Ca^2+^ retention capacity (CRC), and molecular markers associated with anti- and proapoptotic pathways, as described in our previous studies [7, 26, 27].

### 2.3. Mitochondrial Isolation

Mitochondria were isolated as described in our previous studies [17, 28, 29]. All procedures were performed at 4 °C. Hearts were rapidly excised and minced in 4 °C isolation buffer containing in mM: 200 mannitol, 50 sucrose, 5 KH_2_PO_4_, 5 MOPS, 1 EGTA, 0.1% fatty acid free BSA, and protease inhibitors, at pH 7.15 (adjusted with KOH). The tissue suspension was homogenized in a 50 mL centrifuge tube at low speed in cold isolation buffer. The suspension was centrifuged at 8000 *g* for 10 min. The supernatant was collected and centrifuged again at 50,000 *g* for 30 min. After centrifugation, the supernatant was removed and stored for determination of cytosolic proteins. The pellet was resuspended in cold isolation buffer and spun at 850 *g* for 10 min. The supernatant was again collected and re-centrifuged at 8000 *g* for 10 min. Two different types of mitochondria, crude and purified, were isolated. At the end of this last spin (crude), mitochondria were stored in ice and used to assess bioenergetics and CRC. For the purified mitochondria, the supernatant from the last spin was discarded, and the final mitochondrial pellet resuspended. The mitochondria were then purified using 30% percoll in the isolation buffer and then spun at 95,000 *g* for 30 min [26]. The purified mitochondria were kept on ice for western blot experiments. All mitochondrial functional studies were performed at room temperature.

### 2.4. Mitochondrial O_2_ Consumption, Membrane Potential (*ΔΨ*_m_), and Ca^2+^ Retention Capacity (CRC)

Mitochondria isolated at the end of each specified reperfusion time from each experimental group were used to assess the impact of IR with and without treatment on bioenergetics and CRC. The mitochondrial protein content was determined by the Bradford method [7, 26]. Mitochondrial O_2_ consumption was measured using a Clark electrode respirometer (System S200A, Strathkelvin Instruments). The protocol is described in our previous studies [21, 30, 31]. Mitochondria, 0.275 mg from each group, were added into 0.5 mL respiration buffer (in mM: 130 KCl, 5 K_2_HPO_4_, 20 MOPS, 0.1% BSA, pH 7.15) to measure the O_2_ consumption rate during states 2, 3, and 4 respirations. To initiate state 2 respiration, the mitochondrial suspension was energized with 10 mM complex I substrate, Na^+^-pyruvate, and Na^+^-malate mixture (PM; 1 : 1), or the complex II substrate, Na^+^-succinate. State 3 respiration was initiated by adding 250 *μ*M ADP, and state 4 respiration ensued when the added ADP was completely phosphorylated to ATP via oxidative phosphorylation. The respiratory control index (RCI) was calculated as the rate of state 3/state 4 respiration. This value reflects the viability of mitochondria and the tightness of the coupling of oxidative phosphorylation for the different treatment groups during different reperfusion times.

To monitor *ΔΨ*_m_, the cationic fluorescent dye tetramethyl-rhodamine methyl ester (1 *μ*M) (TMRM; Molecular Probes, Eugene, OR) was added to the mitochondrial suspension in respiration buffer as we reported previously [27, 29, 32]. Fluorescence detection representing changes in *ΔΨ*_m_ was monitored using two excitations *λ*_ex_546 and 573 nm and a single emission *λ*_em_590 nm, as described previously [31, 33]. A suspension of 500 *μ*g of mitochondria was added to 1 mL of respiration buffer and energized with 10 mM PM mixture (1 : 1) or with 10 mM succinate. After establishing *ΔΨ*_m_, ADP (250 *μ*M) was added to the mitochondrial suspension to transiently depolarize *ΔΨ*_m_, which reflects state 3 respiration. The *ΔΨ*_m_ repolarizes to baseline after the added ADP is phosphorylated to ATP (state 4 respiration). The time to repolarize was measured for all five groups. At the end of each experiment, 4 *μ*M carbonyl cyanide m-chlorophenyl hydrazone (CCCP), a mitochondrial uncoupling agent, was added to the mitochondrial suspension to fully depolarize *ΔΨ*_m_. The magnitude of the ADP depolarization was compared to the maximal depolarization when CCCP was added.

Our previous studies showed that acute IR leads to increased cytosolic and mitochondrial Ca^2+^ overload and that these effects were mitigated by hypothermia, but not by CP [11, 12]. Ca^2+^ overload could weaken mitochondria and lower their threshold for mPTP opening under stress. To test the vulnerability of mitochondria after IR injury, we monitored isolated mitochondrial tolerance to excess CaCl_2_ pulse challenges after IR ± treatments and in TC. CRC was measured by fluorescence spectrophotometry (Qm-8, Photon Technology Instrument; HORIBA Scientific, Piscataway, NJ). Isolated mitochondria (500 *μ*g) were suspended in a cuvette containing 1 mL of respiration buffer with 0.1 *μ*M calcium fluorescent dye Fura-4F K^+^-salt with dual excitation wavelengths (*λ*_ex_) at 340/380 nm and a single emission wavelength (*λ*_em_) at 510 nm [27, 29]. The mitochondrial suspension in the cuvette was under continuous stirring and was energized with 10 mM PM mixture (1 : 1) or with 10 mM Na^+^-succinate after 60 s at room temperature. Afterwards, mitochondria were challenged with repeated boluses of CaCl_2_ (20 *μ*M) every 90 s, in the presence of approximately 40 *μ*M EGTA (carried over from the isolation buffer), until mitochondria were not able to take up and sequester the added Ca^2+^ or the mPTP opens. The opening of mPTP is indicated by a sharp increase in the extra-matrix-dye fluorescent intensity due to increase Ca^2+^ release into the medium. Mitochondrial Ca^2+^ uptake was determined by the decay of the extra-matrix fluorescent dye (Fura-4F) intensity signal towards baseline, after the addition of boluses of CaCl_2_. A steady-state Ca^2+^ uptake was achieved by the flat response in the curve as mitochondria take up and sequester the added Ca^2+^ [27]. After mPTP opening or no additional Ca^2+^ uptake, CCCP (4 *μ*M) was added to obtain the maximal fluorescence signal based on the total release of the sequestered Ca^2+^.

### 2.5. Western Blot Assays for Hexokinase II (HKII), Phospho-Akt, Bax, and Cytochrome c (Cyt-c) in Cytosolic and Mitochondrial Fractions

At specified reperfusion times, the cytosolic fraction was separated from the mitochondrial fraction by differential centrifugation as described before [7, 17]. Both the cytosolic and mitochondrial fractions were stored at −80 °C until the time of experimentation. We used standard western blotting procedures to assess the levels of targeted anti- and proapoptotic proteins following the determination of the protein content. To verify the purity of the mitochondrial and cytosolic fractions, COX IV was used as the internal housekeeping protein for the mitochondrial fractions, and *β*-tubulin was used as internal housekeeping protein for the cytosolic fractions.

The cytosolic and mitochondrial protein lysates were resolved by SDS-polyacrylamide gel electrophoresis (SDS-PAGE) using a 4%-20% slab gel. After the gel electrophoresis, proteins were transferred onto a polyvinylidene difluoride (PVDF) membrane using the Transblot system (Bio-Rad, Richmond, CA). Membranes were incubated with specific primary antibodies: anti-HKII (rabbit monoclonal, 1 : 1000, Cell Signaling Technology); anti-Bax (1 : 1000 dilution; Cell Signaling); anti-Akt (1 : 1000 dilution; Cell Signaling); anti-pAkt at serine 473 (Ser473; 1 : 1000 dilution; Cell Signaling); anti-Cyt-c (mouse monoclonal, 1 : 1000, Invitrogen); anti-*β* tubulin (rabbit monoclonal, 1 : 1000, Cell Signaling Technology); and anti-COX IV (rabbit monoclonal, 1 : 1000, Cell Signaling Technology) overnight at 4 °C. After washing 3 times, the membranes were incubated with the appropriate secondary antibody conjugated to HRP and then immersed in an enhanced chemiluminescence detection solution (GE Healthcare), and images were taken using the ChemiDoc imaging system (Bio-Rad).

### 2.6. Statistical Analysis

Data are expressed as means ± standard error of the means (SEM). Student-Newman-Keuls' test was used to differentiate the differences within or between groups. If *p* values were significant (*p* < 0.05), one-way ANOVA was selected to analyze the statistically significant differences among the groups. Differences among the means were considered significant when *p* < 0.05 (two-tailed).

## 3. Results

### 3.1. Hypothermia and Cardioplegia Protected Cardiac Mechanical Function and Redox State during IR Injury

**Figure 2:**
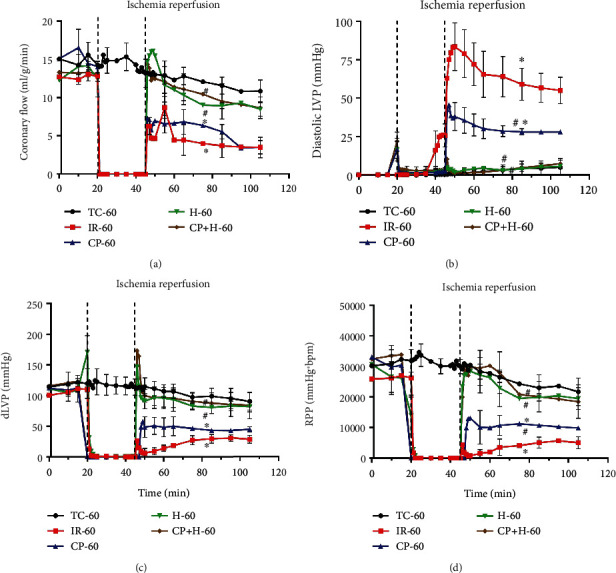
Coronary flow (CF), diastolic LVP (DiaLVP), developed LVP (dLVP), rate pressure product (RPP) at 60 min reperfusion in time control (TC), ischemia reperfusion (IR), cardioplegia (CP), hypothermia (H), and cardioplegia + hypothermia (CP + H) groups. *N* = 6 hearts in each group. Values are mean ± SE.  ^∗^*p* < 0.05, compared to TC group;  ^#^*p* < 0.05 compared to IR group.

The timeline of the experimental protocol is shown in Figure 1. Cardiac functional data were assessed before, during, and at each specified reperfusion time (5, 20, and 60 min) to correlate with mitochondrial function and translocation of signaling molecules to and from mitochondria associated with promotion of cell survival *vs.* cell death, respectively. Baseline functional measurements of coronary flow (CF), diastolic left ventricular pressure (LVP), developed LVP (dLVP), and rate pressure product (RPP), were not significantly different among all five groups (Figure 2). The time control (TC) group showed relative constancy of each variable monitored during a specified perfusion protocol. At reperfusion 60 min, all the functional variables were significantly worsened in the IR alone group. IR + CP did not improve recovery of CF but showed a modest but significant recovery of all LVP indices and RPP when compared to the IR alone group. H and HCP treatments with IR led to almost full recovery of all functional variables after ischemia, as they were not significantly different from the TC at 60 min reperfusion. In this acute IR injury model, adding CP to the 17 °C perfusate did not provide any additional protection against the acute IR injury. The marked increase in diastolic LVP, an index of left ventricular contracture, in the IR group, is likely due to impaired cytosolic Ca^2+^ homeostasis, which would contribute to the diminished recovery of dLVP and RPP (Figure 2). The functional recovery of the four variables at 5 and 20 min reperfusion in the IR + H, CP, and HCP (Supplementary Figure S1) groups is comparable to that found after 60-min reperfusion. Values for *d*LVP/*d*t_max_ and *d*LVP/*d*t_min_, i.e., rates of contractility and relaxation, complemented the data displayed in Figure 2 (Table 1). The IR group had the lowest *d*LVP/*d*t_max_ and *d*LVP/*d*t_min_ (Table 1). IR + H, CP, and HCP groups improved recovery of *d*LVP/*d*t_max_ and *d*LVP/*d*t_min_ at 60 min reperfusion. Moreover, recovery of contractility and relaxation after IR in the H and HCP groups were significantly better than in the IR + CP group. The recovery of *d*LVP/*d*t_max_ and *d*LVP/*d*t_min_ during 5 and 20 min reperfusion were again significantly better in the H and HCP treated hearts (Supplementary Table S1).

NADH and FAD autofluorescence, markers of the mitochondrial redox state, were monitored before, during, and after acute IR. NADH fluorescence signal increased, and FAD fluorescence signal did not change significantly during ischemia when compared to the TC group (Table 2). The increase in NADH signal is likely due to inhibited electron flow along the electron transfer chain (ETC) as we have reported before [12, 13]. The increase in NADH was significantly more pronounced in the H and HCP groups during ischemia than in the IR alone (control) and CP groups. However, redox state was not different among all groups at 20 min reperfusion (Table 2). This indicated that H and HCP treatments elicited a higher (reduced) redox state during ischemia so that reducing equivalents were readily available on reperfusion for oxidative phosphorylation to restore ATP levels. Overall, these results indicated that in this acute IR model, hypothermia-conferred protection, just before ischemia and in early reperfusion, largely by protecting mitochondria function during the period of reperfusion.

### 3.2. Hypothermia and Cardioplegia Improved Respiratory Control Index (RCI), Mitochondrial Membrane Potential (*ΔΨ*_m_), and Ca^*2*+^ Retention Capacity (CRC) after IR Injury

**Figure 3:**
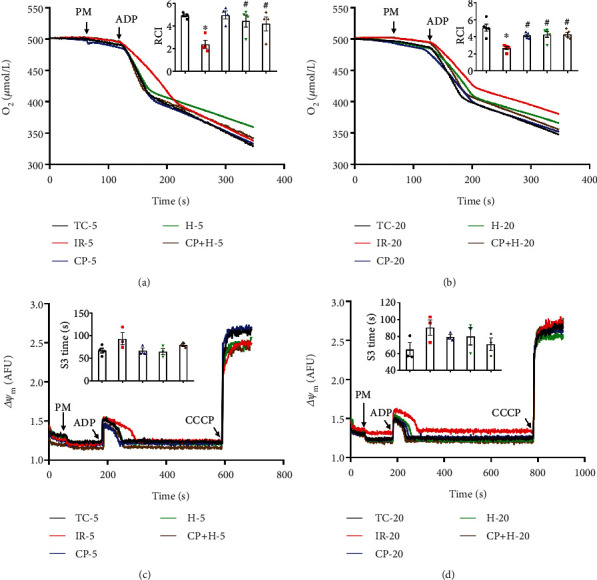
Representative traces of O_2_ consumption rates (insets: average respiratory control index (RCI)) at 5 (a) and 20 min (b) reperfusion periods in time control (TC), ischemia reperfusion (IR), cardioplegia (CP), hypothermia (H), and cardioplegia + hypothermia (CP + H) groups. Change in membrane potential (*ΔΨ*_m_) (insets: average time (S3 time (sec)) for repolarization after ADP-induced depolarization (state 3)) at 5 (c) and 20 min (d) reperfusion periods in experimental groups. Mitochondria were energized with Na^+^-pyruvate and Na^+^-malate (PM; complex I substrate). *N* = mitochondria from 3 to 4 hearts in each group. The bar graphs show mean ± SE.  ^∗^*p* < 0.05 compared to TC group;  ^#^*p* < 0.05 compared to IR group.

**Figure 4:**
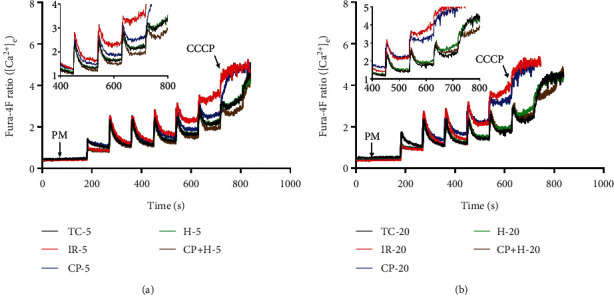
Representative traces of mitochondrial calcium retention capacity (CRC) at 5 (a) and 20 min (b) reperfusion periods in time control (TC), ischemia reperfusion (IR), cardioplegia (CP), hypothermia (H), and cardioplegia + hypothermia (CP + H) groups. The insets are representative traces at later time points that show in greater details the differences in the kinetics of mitochondrial Ca^2+^ uptake during the CaCl_2_ pulse challenges until mitochondria stopped taking Ca^2+^. CCCP, the mitochondrial uncoupler, was given after mitochondria stopped taking the added Ca^2+^ to unload all the Ca^2+^ sequestered during bolus additions. Mitochondria were energized with Na^+^-pyruvate and Na^+^-malate (PM; complex I substrate).

Changes in bioenergetics, including the state 3 over state 4 respiration ratio, i.e., RCI (Figures 3(a) and 3(b)) and *ΔΨ*_m_ (Figures 3(c) and 3(d)), and the ability of mitochondria to take up and retain added Ca^2+^ (CRC) (Figure 4) were assessed in TC, IR alone, and IR + H, CP, or HCP treated hearts after isolating mitochondria at the specified reperfusion times of 5 and 20 min. We examined mitochondrial function at these specified times based on our previous reports that showed deleterious effects that contribute to later reperfusion injury that occurs during early reperfusion [17, 25]. Mitochondrial O_2_ consumption and RCI were monitored in mitochondria energized with the complex I substrate PM or the complex II substrate succinate. In mitochondria energized with PM, O_2_ consumption was significantly depressed in the IR group at 5 and 20 min reperfusion; H, CP, and HCP treatments each restored states 3 and 4 O_2_ consumption rates and RCI to values not significantly different from the TC group. However, states 3 and 4 O_2_ consumption rates and RCIs were not altered significantly in mitochondria energized with the complex II substrate in any group (Supplementary Figure S2).

The time to repolarize *ΔΨ*_m_ (state 4 respiration) following ADP-induced depolarization (state 3 respiration) was assessed by TMRM fluorescence at 5 and 20 min reperfusion (Figures 3(c) and 3(d)). State 2 and state 4 *ΔΨ*_m_ were similar for both substrates in all groups. After adding ADP, there was a rapid, partial, and reversible *ΔΨ*_m_ depolarization in each group. IR-5 (Figure 3(c)) and IR-20 (reperfusion at 5 and at 20 min) (Figure 3(d)) groups showed a significant delay in *ΔΨ*_m_ repolarization compared to the treatments and their respective TC groups, with both substrates (Figures 3(c) and 3(d) and Supplementary Figures S2(c) and S2(d)). The times for *ΔΨ*_m_ repolarization following ADP-mediated *ΔΨ*_m_ depolarization were not significantly different among IR + H, CP or HCP groups from the TC groups at the 5 and 20min reperfusion with PM as the substrate. The duration of *ΔΨ*_m_ depolarization was longer for the complex II substrate succinate than for the complex I substrate PM after IR-5 and IR-20 (Figures 3(c) and 3(d) and Supplementary Figure S2). Recovery of *ΔΨ*_m_ after ADP-induced depolarization was impaired in the IR only group. H, CP, and HCP treatments with IR exhibited *ΔΨ*_m_ repolarization like that in the TC groups.

Mitochondrial CRC was assessed by uptake and release of Ca^2+^ into the respiratory buffer containing 40 *μ*M EGTA using Fura-4 fluorescence after 5 min (Figure 4(a)) and 20-min (Figure 4(b)) reperfusion. The inset in each panel is an expanded view of the Ca^2+^ uptake and retention profiles for the last several CaCl_2_ pulses just before mPTP opened, i.e., when mitochondria stop taking up Ca^2+^. Note that with PM as substrate, isolated mitochondria from the IR + H and IR + HCP hearts took up and retained Ca^2+^ better than mitochondria isolated from the IR only hearts (inset, Figures 4(a) and 4(b)). There was no significant difference in CRC between IR + H and IR + HCP groups, which is consistent with the results on the cardiac functional recovery. Although CP alone provided a modest improvement of cardiac function and mitochondrial bioenergetics, it did not significantly improve CRC compared to the IR only group. This effect of CP is consistent with our previous study [11], which showed that in the *ex vivo* heart, CP-induced protection against acute IR injury did not reduce mitochondrial Ca^2+^ overload despite the modest recovery of function. Our results show that hypothermia after reperfusion better preserved mitochondria Ca^2+^ handling during excess CaCl_2_ challenges, which agrees with its mitigating effect on mitochondrial Ca^2+^ overload in the acute *ex vivo* IR model [13]. There was no difference among the groups in the CRC when mitochondria were energized with the complex II substrate, succinate (Supplementary Figure S3). Overall, these results indicated that hypothermia is more protective with PM used as the substrate.

### 3.3. Hypothermia Promoted HKII Binding to Mitochondria after IR Injury

**Figure 5:**
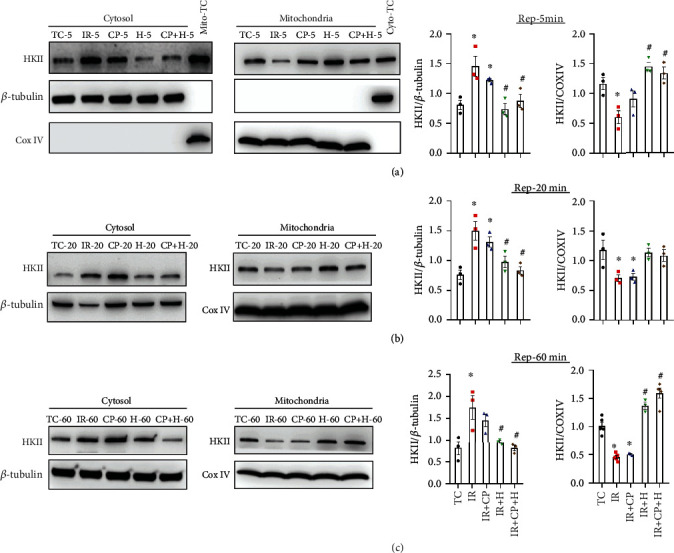
Western blot analysis of hexokinase II at 5, 20, and 60 min reperfusion periods in time control (TC), ischemia reperfusion (IR), cardioplegia (CP), hypothermia (H), and cardioplegia + hypothermia (CP + H) groups. *N* = mitochondria from 3 to 4 hearts in each group. Values are mean ± SE.  ^∗^*p* < 0.05 compared to TC group;  ^#^*p* < 0.05 compared to IR group.

We tested next whether cardioprotection against acute IR injury afforded by IR + H, CP, and HCP is mediated, in part, by increased HKII translocation/binding to mitochondria at 5, 20, and 60 min reperfusion. Western blotting of HKII bound to mitochondria and HKII expression levels in the cytosolic fraction after IR alone showed significant dissociation of HKII binding from mitochondria, while showing increased levels of HKII in the cytosolic fraction (Figure 5). IR + H and IR + HCP groups showed significant binding of HKII to mitochondria and, correspondingly, decreased HKII levels in the cytosolic fraction at 5, 20, and 60 min reperfusion when compared to IR alone (control) hearts. There was no significant difference in mitochondrial HKII binding between H and HCP groups at 5, 20, and 60 min. Of note, the binding of HKII to mitochondria in the HCP group at 60 min reperfusion was significantly greater than in the TC group (Figure 5(c)). The data suggested that H and HCP maintained mitochondria-HKII association and improved mitochondria function as contributing factors in the robust recovery of cardiac function after IR. Just as CP alone provided only modest protection of cardiac function against IR injury, there was no significant HKII binding to mitochondria or altered HKII levels in the cytosolic fraction at 5, 20, or 60 min reperfusion in the CP group.

### 3.4. Hypothermia Increased Akt (Protein Kinase B) Phosphorylation (pAkt) at Ser473 after IR Injury

**Figure 6:**
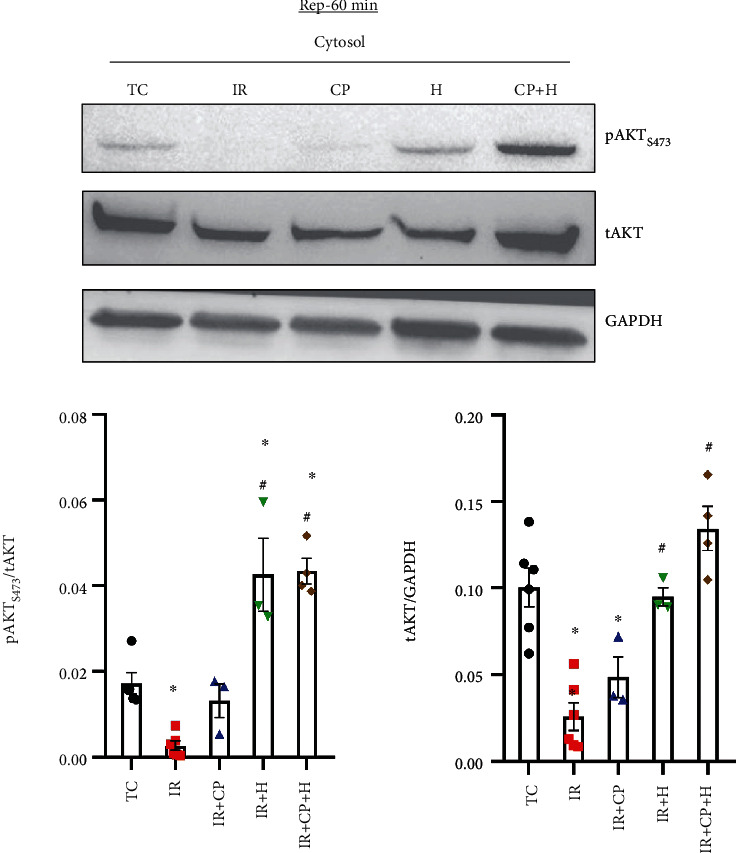
Western blot analysis of total Akt (tAkt) and phosphor-Akt at Ser 473 (pAkt) at 60-min reperfusion in time control (TC), ischemia reperfusion (IR), cardioplegia (CP), hypothermia (H), and cardioplegia + hypothermia (CP + H) groups. *N* = mitochondria from 3 to 5 hearts in each group. Values are mean ± SE.  ^∗^*p* < 0.05 compared to TC group;  ^#^*p* < 0.05 compared to IR group.

Because we observed that H and HCP treatments markedly maintained HKII binding to mitochondria (Figure 5), we determined next whether phosphorylated (p) Akt levels correspondingly increased in the IR + H, CP, and HCP groups. Since HKII binding to mitochondria was sustained for up to 60-min reperfusion in H and HCP treated hearts, we examined the phosphorylation of Akt at Ser473, which is associated with Akt activation an upstream activator of HKII [34, 35] in the cytosolic fraction after 60-min reperfusion (Figure 6). CP treatment did not increase cytosolic pAkt levels compared to the IR group. The diminished levels of pAkt in the IR group are consistent with the reduced HKII binding to mitochondria; in the CP group, there was a modest increase in pAkt, but this increase was not associated with increased HKII-mitochondria binding. In the H and HCP groups, there was a marked increase in pAkt, which is consistent with maintained HKII binding to mitochondria. There was a significant decrease in total (t) Akt (tAkt) expression in the IR and CP groups, compared to the TC group, indicating that IR itself reduces the levels of Akt leading to a decline in the tAkt level. H and HCP preserved tAkt levels when compared to the TC group. Altogether, these results indicated that cardioprotection afforded by H and HCP was mediated, in part, by increased levels of cytosolic pAkt, which could contribute to the maintenance of HKII binding to mitochondria. In contrast, the modest cardioprotection afforded by CP treatment was independent of the Akt-HKII signaling pathway.

### 3.5. Bax Binding Was Not Altered by Hypothermia or Cardioplegia after IR Injury

We next monitored Bax, a proapoptotic protein, in mitochondrial and cytosolic fractions to assess whether H, CP, or HCP treatment alters Bax binding to mitochondria to confer protection. We found that the levels of Bax in the mitochondrial and cytosolic fractions were not significantly different among the five groups, regardless of the reperfusion time (Supplementary Figure S4). Interestingly, the H and HCP-induced increases in HKII binding to mitochondria did not preclude Bax binding to mitochondria, as has been reported during ischemic stress [36].

### 3.6. Hypothermia Decreased mitochondrial Cytochrome c (Cyto-c) Release after IR Injury

**Figure 7:**
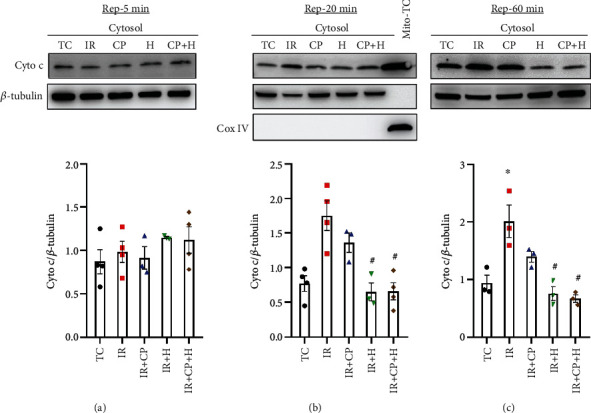
Western blot analysis of cytosolic cytochrome c (Cyto-c) release into the cytosol at 5 (a), 20 (b), and 60 (c) min reperfusion periods in time control (TC), ischemia reperfusion (IR), cardioplegia (CP), hypothermia (H), and cardioplegia + hypothermia (CP + H) groups. *N* = mitochondria from 3 to 4 hearts in each group. Values are mean ± SE.  ^∗^*p* < 0.05 compared to TC group;  ^#^*p* < 0.05 compared to IR group.

We then correlated the timing of HKII bound to mitochondria with Cyto-*c* release in the cytosol, a marker of mPTP opening or mPTP-independent OMM permeability, after IR injury. At 5 min reperfusion, there was no significant difference in cytosolic Cyto-*c* content among the groups (Figure 7(a)). However, at 20 and 60 min reperfusion, there was a significant increase in Cyto-*c* release in the IR group. H and HCP treatments significantly reduced the IR-induced cytosolic Cyto-*c* levels to levels not significantly different from TC hearts (Figure 7(b) and 7(c)). The sustained increase in HKII binding at 20 and 60 min reperfusion was associated with reduced Cyto-*c* release into the cytosol and improved cardiac function after IR. Since HKII translocation to mitochondria was already detected at 5-min reperfusion, these data appear to confirm that HKII binding to mitochondria before Cyto-*c* release is a time-dependent signaling event preceding release of the apoptotic agent. CP did not significantly reduce Cyto-*c* release induced by IR when compared to the IR group, suggesting that the modest CP-mediated protection is independent of these signaling molecules that converge on (HKII) or emanate (Cyto-*c*) from mitochondria. Together, these experiments show hypothermia as preserving mitochondria integrity and function, in part, by conserving HKII-binding to mitochondria and by reducing apoptotic signaling, culminating in the full recovery of cardiac function after acute IR.

## 4. Discussion

Hypothermia with and without CP has been widely used in animal models of IR and in clinical procedures to provide cardioprotection [3, 6, 37]. We have previously reported that hypothermia and CP preserve cardiac function and protect against tissue infarction [10–13, 38, 39]. However, there remains a lack of knowledge on time-dependent changes in mitochondrial functions controlled by signaling molecules during the early and later reperfusion periods in acute IR injury. In the present study, our goal was to determine how hypothermia and CP modulate signaling molecules to protect the myocardium during increasing post-ischemic times of reperfusion and to unravel the role of signaling mechanisms that converge at the mitochondria in providing protection.

Our findings are that (a) acute IR injury is independent of Bax because there was no difference in Bax translocation in the IR group compared to the treatment groups; (b) the modest CP-mediated cardioprotection against acute cardiac IR injury is independent of HKII binding to mitochondria; (c) the robust H- and HCP-mediated cardioprotection are mediated, at least in part, by promoting HKII, a pro-survival signaling molecule, binding to mitochondria, likely, via increased pAkt, an upstream signaling molecule; (d) release of the proapoptotic protein Cyto-*c*, which was first observed after 20 min reperfusion and persisted during later reperfusion, was blunted by H and HCP, but not by CP; (e) H, CP, and HCP treatments improved mitochondrial respiration and *ΔΨ*_m_, but only H and HCP better maintained the capacity of mitochondria to take up and retain Ca^2+^ during pulse application of exogenous CaCl_2_; and (f) NADH was more reduced during ischemia by H and HCP. This latter effect may reflect less damage to complex I of the ETC as suggested using modulators of complex I [7, 18, 40]. Overall, our study demonstrates that molecular mechanisms that converge on mitochondria underlie hypothermia-mediated cardioprotection, whereas CP-induced protection appears to be less dependent on mitochondria because it did not prevent Cyto-*c* release in a HKII-dependent manner. These differences in evoking mitochondrial signaling mechanisms appear to parallel the more effective protection afforded by H and HCP compared to CP alone.

### 4.1. Hypothermia, Cardioplegia, and Cardioplegia + Hypothermia Differentially Protects the Myocardium from Acute IR Injury

Myocardial reperfusion injury increases as the duration of ischemia increases [5]. Reperfusion injury is a process that causes metabolic alterations like Ca^2+^ overload, lactic acidosis, and reduced ATP synthesis, which lead to activation of signaling pathways and promote mPTP opening to ultimately cause apoptotic and necrotic cell death [1, 3, 15]. Reperfusion is a double-edged sword; on the one hand, it activates endogenous events that salvage viable myocytes, while on the other hand, it exacerbates the ischemic injury, manifested as reperfusion injury.

Hypothermia is a highly effective cytoprotective approach because it can be utilized temporarily and because the reduction in myocardial metabolism is reversible with rewarming. The basic mechanisms underlying hypothermic cardiac protection are the overall reduction in enzymatic activity, most importantly those reactions that require ATP consumption [41]. During cold ischemia, hypothermia protects by attenuating the mitochondrial ATP consuming processes, thus increasing ATP reserve; in this way, on warm reperfusion, mitochondrial respiration becomes less dysfunctional, and ATP becomes more readily regenerated for restoring ionic homeostasis and cardiac contractile and relaxant function. However, prolonged hypothermia, per se, can induce ROS production by slowing the activity of antioxidant enzymes, which could lead to impaired functional recovery because of greater ROS emission [12, 41].

A depolarizing, cold CP solution remains the standard-of care for inducing diastolic arrest during extracorporeal heart perfusion to facilitate cardiac surgery [42]. CP is designed to rapidly cease cardiac contractions and so reduce the metabolic rate and decrease O_2_ demand [43]. But warm CP itself has little protective effect other than to induce cardiac arrest to facilitate surgical repairs [44]. Moreover, CP can contribute to post-cardioplegic injury by inducing dysrhythmias during reperfusion [45]. Although both CP and H individually have limitations, the combination HCP appears a better approach to mitigate their individual shortcomings [46].

We proposed to determine whether H, CP, and HCP protection involved signaling pathways during earlier and later reperfusion that would underlie improved mitochondrial bioenergetics and provide protection against acute IR injury. As expected, normothermic acute IR injury led to a predictable decrease in cardiac function during reperfusion (Figure 2 and Supplementary Figure S1) with no further improvement after the initial 5 min of reperfusion. This observation bolsters our previous findings that early events at reperfusion, e.g., cytosolic and mitochondrial Ca^2+^ overload, are critical determinants of cardiac functional recovery during later reperfusion. The protection by H and HCP support this notion because these interventions given for only 5 min before and for 2 min immediately after reperfusion (Figure 1) largely enhanced protective mediators or suppressed deleterious factors that improved or compromised cardiac function, respectively, on reperfusion. We observed that the increase in diastolic contracture, which correlates with cytosolic and mitochondrial Ca^2+^ dysregulation [7, 9, 11, 12], possibly due to diminished mitochondrial ATP production, begins during ischemia and continues throughout reperfusion. In the IR group, diastolic contracture was markedly enhanced during later ischemia and during the entire reperfusion period; in the CP group, the diastolic contracture was significantly reduced when compared to the IR group, but remained higher than the TC group. This suggests that CP when compared to the IR group promoted a greater supply and utilization of ATP to restore cellular ionic homeostasis.

In contrast, H and HCP treatments completely prevented the diastolic contractures seen during ischemia and reperfusion to levels not significantly different from the TC group. The decrease in diastolic contracture likely reflects even better restoration of ATP production and better phasic Ca^2+^ handling in the H and HCP groups *vs.* the CP group. The lack of diastolic contracture was associated with marked improvements in cardiac contraction and relaxation that was equivalent to that found in the TCs (Figure 2 and Supplementary Figure S1; Table 1 and Supplementary Table S1). In our previous studies with acute ischemia and longer reperfusion time, we reported that CP and H provided varying degree of protection [11, 13]. In a cold, 2 h ischemia model, we found that HCP was more effective than H alone [10]. This is because hypothermia, even at increasingly lower temperatures, does not provide complete protection as the time of ischemia is extended [13]. For our current study, we then investigated several molecular mechanisms that might underlie the differences in H and CP protection following acute ischemia followed by various reperfusion times at 5, 20, and 60 min.

### 4.2. Hypothermia Best Maintains Mitochondrial Bioenergetics and Ca^2+^ Retention Capacity (CRC) during Reperfusion

Mitochondrial dysfunction in acute IR injury is a critical determinant of cell death because mitochondria are crucial regulators of cell life and death [8]. Mitochondria generate most of the ATP in cardiomyocytes to maintain cellular and mitochondrial ionic homeostasis. In cardiomyocytes, mitochondria produce the reducing equivalents NADH and FADH_2_ in the TCA cycle, required for oxidative phosphorylation. NADH and FADH_2_ are oxidized by complex I and complex II enzymes, respectively. Because the redox state (NADH/FAD) is dependent on the transfer of electrons (oxidation), monitoring NADH (reduced) and FAD (oxidized) online in the *ex vivo* perfused heart is a useful method to assess the state of mitochondrial redox state before, during, and after ischemia.

During ischemia, due to the decrease in O_2_ delivery, an increase in NADH and a corresponding decrease in FAD represent a more reduced mitochondrial redox state [9]. In this study, the increase in NADH during ischemia (Table 2) is consistent with our previous observations [10, 13]. Our results here demonstrate that at the end of ischemia (25 min), H and HCP treatments increased NADH more than did the CP treatment or IR alone, but there was no significant change in FAD; this likely indicates that complex II was less impacted than complex I during the acute ischemia. These observations also suggest that H and HCP preserve mitochondrial function in part by protecting complex I, which would be energetically beneficial for the myocardium on reperfusion. Therefore, the decline in NADH during ischemia in the IR group compared to the H and HCP groups could be attributed to damage to complex I [7, 18, 40].

This notion is consistent with our previous findings [18] in which we reported that acute, warm IR led to a decrease in complex I activity, in part, because of oxidative damage to cardiolipin, a major inner mitochondrial membrane (IMM). This damage impairs mitochondria ability to maintain *ΔΨ*_m_ and to provide a sustained energy supply on reperfusion [18]. By maintaining a robust redox state during ischemia, H and HCP preserved ETC function, which is necessary for ATP production, to maintain cellular ionic homeostasis during reperfusion, and so to abrogate diastolic contracture (Figure 2). Our mitochondrial functional results are consistent with an *in vivo* report showing that hypothermia attenuates oxidative stress by protecting the respiratory enzymes in a pig cardiac arrest model [47].

To further verify the observed improvement in bioenergetics at the intact organ level by H and HCP after IR, we isolated mitochondria at 5 and 20 min reperfusion times and monitored the rate of O_2_ consumption, RCI and *ΔΨ*_m_. We focused on these reperfusion times because our previous studies showed that the most deleterious consequences of IR (cytosolic and mitochondrial Ca^2+^ overload and mitochondrial ROS production) are greatest during early reperfusion [7, 9, 11]. The mitochondrial suspension was energized with complex I or complex II substrates. Unlike IR alone, H, CP, and HCP treatments maintained states 3 and 4 respiration and restored RCI near to that of the TC group at 5 and 20 min reperfusion. These results confirm that H and HCP directly modulate bioenergetics to preserve mitochondrial function very early in reperfusion (Figure 2 and Supplementary Figure S1). The improved RCI by H and HCP corresponds to the improved repolarization of *ΔΨ*_m_ (fast state 3 respiration), which suggests a more coupled oxidative phosphorylation. Interestingly, CP also improved RCI and *ΔΨ*_m_ repolarization in isolated mitochondria; however, unlike H and HCP, the protection did not contribute to a significant improvement in cardiac function. This suggests that the modest CP-mediated cardioprotection is dependent on more than just restoration of mitochondrial function.

Mitochondrial Ca^2+^ is vital to the regulation of mitochondrial bioenergetics; but in excess, it is a key instigator of cell death. One of the driving forces for mitochondrial Ca^2+^ uptake is a fully charged *ΔΨ*_m_. During acute IR, hypothermia reduces mitochondrial Ca^2+^ overload and improves bioenergetics [13]. Ca^2+^ uptake, along with decreased Ca^2+^ egress, during IR tends to depolarize *ΔΨ*_m_ by diminishing the charge gradient and limiting matrix Ca^2+^ sequestration. Unlike hypothermia, we reported that CP-mediated cardioprotection during acute ischemia did not limit mitochondrial Ca^2+^ overload [11], possibly making mitochondria more susceptible to mPTP opening compared to hearts treated with H or HCP. The monitoring of CRC, an *in vitro* surrogate for assessing mitochondrial Ca^2+^ handling, was used to determine the susceptibility of mitochondria to mPTP opening following acute IR stress, as previously reported [48]. We found that in cardiac mitochondria isolated after reperfusion, IR + H and IR + HCP treatment allowed for a more robust uptake and sequestration of Ca^2+^ after adding exogenous CaCl_2_ boluses than did IR and IR + CP (Figure 4). These isolated mitochondrial studies indicate that hypothermia is more protective in preserving mitochondrial Ca^2+^ handling and thereby lowering matrix free Ca^2+^ overload during IR stress, as we have reported in the *ex vivo* perfused heart [11, 13].

Interestingly, all treatments showed a similar CRC with the complex II substrate succinate, again confirming that impaired complex I during IR contributes in part to the impaired mitochondrial bioenergetics (*ΔΨ*_m_) and increases the susceptibility to *ΔΨ*_m_ dissipation during exogenous CaCl_2_ pulse challenges. Under physiological conditions, it is the NADH derived from pyruvate (complex I) oxidation that establishes most of the proton motive force. This is because unlike complex I, complex II does not pump protons with the electron transfer. Therefore, our results suggest that H and HCP preserve complex I function, maintain a polarized *ΔΨ*_m_, enhance tolerance to excess Ca^2+^, and improve mitochondrial respiration during reperfusion. Thus, enhanced capacity of mitochondria to take up and sequester the excess Ca^2+^ accounts, at least partially, for the improved function in the H and HCP groups.

### 4.3. Hypothermia Promotes HKII Binding to Mitochondria and Protects Against Acute Ischemia and Reperfusion Injury

As mentioned above, a profound decrease in cellular metabolism is an important way by which H and CP protect the cell from IR stress. However, the molecular mechanisms and timing by which hypothermia and CP provide protection are not well established. As mediators of the first enzymatic step in glucose metabolism, hexokinases (HKs) orchestrate a variety of metabolic events and modulate cell death processes by directly interacting with mitochondria [6, 49–54], in part, via the voltage dependent anion channel 1 (VDAC), a putative regulatory component of the mPTP [6, 55]. HKII, a cardiac isoform of HK, binds to mitochondria with high affinity at the OMM “contact site” where it interacts with VDAC1. HKII bound to mitochondria is antiapoptotic [54], and a decrease in HKII binding to mitochondria makes cells sensitive to apoptotic stimuli [49]. HKII binding to heart mitochondria has also been implicated in resistance to reperfusion injury by inhibiting or desensitizing mitochondria to early mPTP opening [37]. HKII dissociation from heart mitochondria during ischemia, and the extent of this dissociation, correlates with the infarct size on reperfusion [56]. Thus, we investigated whether acute IR injury leads to decreased HKII binding to mitochondria and if H, CP, or HCP modulates this binding to mitigate cardiac dysfunction following acute IR injury.

Our previous study demonstrated that HKII dissociation from mitochondria worsened IR injury and that interventions that maintain its binding to mitochondria improved cardiac function on reperfusion [26]. HKII binding to mitochondria has also been shown to improve apposition at contact sites between the IMM and outer mitochondrial membrane (OMM) and to maintain the cristae structure [52]. In support of the notion of improved contact between IMM and OMM, we reported recently that HKII binding to mitochondria increased the interaction between VDAC1 in the OMM and adenine nucleotide translocator (ANT) in the IMM [26]. Stabilization of the contact site has been reported to reduce OMM permeabilization and to improve the transfer of ADP/ATP and metabolites across the membranes, which is critical for mitochondrial homeostasis and function [53].

In the current study, we showed that H and HCP treatments maintained HKII binding to mitochondria at 5min reperfusion and that the binding persisted for up to 60-min reperfusion. The early translocation of HKII to mitochondria, or its lack of dissociation during hypothermia, suggests that HKII binding could impede the destabilization of mitochondrial membranes and Cyto-c release and protect against vulnerability to cell death [35, 37, 52, 57]. It is also possible that the sustained HKII binding maintains the contact sites between OMM and IMM to enhance bioenergetic activity [26]. Increased HKII binding to mitochondria by H and HCP facilitates glucose entry into the glycolytic process [52], thereby increasing pyruvate production and oxidative phosphorylation. With improved bioenergetics (i.e., reduced redox state, improved RCI, and normalized *ΔΨ*_m_), the increased ATP production in the H and HCP groups leads to better ionic homeostasis and abrogation of diastolic contracture. Overall, promoting the association of HK with mitochondria, as in hypothermia, tends to protect mitochondria against IR injury, while preventing association of HK to mitochondria leads to greater IR injury and cell death [35].

The CP treatment did not reverse the effect of IR-induced dissociation of HKII from mitochondria; we posit that this could impede glycolysis, concomitantly affecting bioenergetics, and contribute only modest protection against the diastolic contracture and contractile effort (e.g., RPP) compared to H and HCP treatment (Figure 2 and Supplementary Figure S1; Table 1). This suggests that, at least in our model, CP does not offer as adequate a protection during IR as does hypothermia because CP did not maintain binding of HKII to mitochondria that would lead to a robust protection.

### 4.4. Hypothermia Increases Cytosolic pAkt Levels during Reperfusion

Akt is a pro-survival kinase that mediates mitochondrial protection, and its phosphorylation (pAkt) results in its activation and rapid translocation to cellular compartments where it phosphorylates pro- (inactivates) or anti-apoptotic (activates) proteins, like HKII [6, 37]. Indeed, cells that are devoid of the gene for Akt are impaired in their ability to bind HKII to mitochondria [53]. Increased association of HKII with mitochondria as mediated by Akt has been implicated in conferring cardioprotection [50, 54]. The Akt pathway is reported to be involved in protection by hypothermia against cardiac arrest and hemorrhagic shock [16, 58].

The role and interaction of pAkt-HKII during hypothermia in mediating protection during IR has not been well explored. Since HKII is a substrate for Akt and H and HCP increased HKII binding to mitochondria during reperfusion, we sought to know whether total Akt and active Akt, i.e., pAkt expression, are associated with the increase in HKII bound to mitochondria. We found that IR injury caused marked decreases in the levels of total Akt and pAkt proteins, potentially contributing to the decline in Akt-HKII signaling. Reperfusion with H and HCP treatment prevented IR-induced Akt loss and resulted in an overall increase in pAkt, which was associated with an increase in HKII binding to mitochondria. In the IR group, and to a lesser extent the CP group, the compromised RCI, poor CRC, and impaired recovery of cardiac function could be attributed, in part, to the decrease in tAKT and pAkt levels, as they are signaling molecules upstream of HKII.

### 4.5. Bax Binding to Mitochondria Is Unaltered by Hypothermia and Cardioplegia during Acute Ischemia and Reperfusion Injury

The translocation of the proapoptotic protein Bax to mitochondria, which leads to its oligomerization during cardiac stress, including IR, contributes to cell damage via OMM permeabilization [59]. HKII bound to mitochondria prevents Bax translocation and confers protection against OMM permeabilization [59]. Unexpectedly, the increase binding of HKII to mitochondria by H and HCP did not preclude Bax binding to mitochondria after the acute IR. Bax has been shown to be essential for OMM permeabilization [60] independent of mPTP opening. A plausible mechanism for this permeabilization is the binding of Bax to mitochondria during oxidative stress and/or Ca^2+^ overload [37, 61]. Bax can permeabilize the OMM, in part, by homo-oligomerization or hetero-oligomerization with VDAC1 or other proapoptotic proteins [6, 8, 37], which could lead to Cyto-*c* release to the cytosol and the trigger of cell death. In our study, we observed similar Bax binding to mitochondria on reperfusion in all treatment groups (Supplementary Figure S4) regardless of Cyto-*c* release (Figure 7). This indicates that in our model of acute IR injury, Cyto-*c* release in the IR alone and IR + CP group was not mediated via Bax permeabilization of the OMM. After H and HCP treatment, the total abrogation of Cyto-*c* release and the full recovery of mitochondrial bioenergetics and cardiac function (Figures 2, 3, and 4) ascertain that Bax binding to mitochondria had no effect in our acute IR model. This observation is consistent with a recent study, in which Lahnwong et al. [62] reported that Bax levels following an acute IR in rats were not significantly different between the treatment groups and the IR group. Moreover, we reported recently [57] that oxidative stress-induced cell death, due to exposure to rotenone, a complex I inhibitor, in VDAC1^−/−^ H9c2 cells vs. WT H9c2 cells, was not mediated via a Bax signaling pathway. Yenari et al. [63] reported similarly that mild hypothermia did not alter Bax expression and translocation to mitochondria in protecting the brain from ischemic injury.

Taken together, these observations suggest that Bax translocation to mitochondria may not be a requisite for OMM permeabilization in our acute IR model or that it may require association with other proapoptotic proteins/activators (e.g., Bak and tBid) to induce OMM permeabilization [53, 64]. It is plausible that in our acute IR model, these activator proteins were not recruited to mitochondria to engender Bax-mediated OMM permeabilization. Consistent with this idea, it was suggested that Bax is not necessary for OMM permeabilization and that other mechanisms may be involved [53].

We further postulate that Cyto-*c* release in the IR and CP groups independent of Bax could be attributed to mPTP opening or destabilization of mitochondrial membranes [64], which could be due to oxidative stress and or Ca^2+^ overload, as we reported previously [7, 11–13, 25, 32, 33]. Thus, H and HCP-induced increase in HKII binding to mitochondria may prevent mPTP opening or stabilize the OMM/IMM contact sites. In this way, apoptosis is attenuated because of more normalized *ΔΨ*_m_, better Ca^2+^ handling, a more tightly coupled RCI, and overall, a more efficient mitochondrial function.

### 4.6. Hypothermia Blunts Cytochrome c Release from Mitochondria during Reperfusion after Ischemia

Cyto-*c* is a labile protein in the mitochondrial intermembrane space. It is critical in the shuttle of electrons in the ETC, and it is also a key scavenger of mitochondrial ROS [19, 37]. In a previous study, we reported that Cyto-*c* release following acute IR was time dependent, with the greatest release into the cytosol at 60 min reperfusion [7]. These results are congruent with our current observations, which show more Cyto-*c* release in the IR group at 20 and 60 min reperfusion (Figure 7). As discussed above, Cyto-*c* release has been associated with increased permeabilization of the OMM. This could be by translocation of proapoptotic proteins (Bax/Bak), VDAC homo-oligomerization, or mPTP opening [37, 55]. Furthermore, undocking of HKII from mitochondria leads to Cyto-*c* release [51] consistent with our results. Cyto-*c* release into the cytosol could contribute to mitochondrial dysfunction that triggers apoptosis and cell death and poor cardiac function during reperfusion.

We monitored Cyto-*c* levels in the cytosol to determine if CP- or H-mediated protection is caused by inhibiting or reducing mitochondrial Cyto-*c* release in a time dependent manner on reperfusion. H and HCP blocked Cyto-*c* release and preserved mitochondrial membrane integrity and bioenergetics and led to improved cardiac function on reperfusion. The blocking of Cyto-*c* release was preceded by enhanced HKII binding to mitochondria in H and HCP groups. Overall, these biochemical and metabolic changes that occurred within the first few minutes of reperfusion were determinants of mitochondrial and cardiac functional outcomes throughout the reperfusion period.

## 5. Conclusions

**Figure 8:**
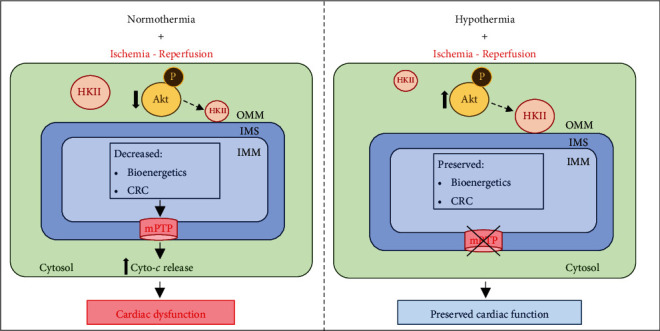
A schematic representation of the proposed molecular events associated with hypothermia mediated cardioprotection against ischemia reperfusion (IR) injury. Hypothermia exerts the cardioprotection by maintaining HKII binding to mitochondria through Akt signaling and, subsequently, delays mitochondrial permeability transition pore (mPTP) opening and decrease the cytochrome *c* (Cyto-*c*) release to preserve mitochondrial bioenergetics and calcium retention capacity (CRC), and hence contributes to cardiac survival and function improvement. OMM, outer mitochondrial membrane; IMS, intermembrane space; IMM, inner mitochondrial membrane.

Hypothermia (H), and to a lesser extent cardioplegia (CP), has been shown to be cardioprotective against IR injury. Our study offers some new mechanistic insights for this by demonstrating the following: (a) H affords better protection of the heart than CP by preserving mitochondrial functional integrity via signaling pathways that converge on mitochondria; (b) H-induced protection is associated with preservation of Akt and increased levels of activated cytosolic pAkt and its substrate HKII binding to mitochondria; (c) the modest cardioprotection afforded by CP is not mediated by the pAkt-HKII signaling pathway; (d) in this acute IR model, HCP mediated protection is not distinct from hypothermia alone, which suggests that the mechanisms examined are similar and hypothermia-mediated protection with or without CP was mostly targeted to mitochondria; (e) the acute IR injury model is independent of Bax binding to mitochondria; (f) H, but not CP, attenuates/inhibits Cyto-c release and thereby preserves *ΔΨ*_m_ and mitochondrial capacity to sequester Ca^2+^ after CaCl_2_ pulse challenges. Collectively, activating the Akt-HKII pathway on reperfusion and the convergence of this signaling mechanism on mitochondria may be, at least in part, an underlying factor that makes hypothermia more efficacious in protecting the heart against the acute IR injury. This protection maintains the integrity of mitochondria by preserving complex I and Cyto-*c*, independent of Bax binding to mitochondria. In closing, this study shows that the robust protection against acute IR injury by hypothermia is mediated, in part, via mitochondria specific events, including Akt-HKII signaling and, concomitantly, decreased Cyto-*c* release from mitochondria into cytosol (Figure 8). In contrast, the modest protection by CP was less mitochondria-targeted and did not involve the Akt-HKII protective signaling and the blunting of mitochondrial Cyto-*c* release. Thus, cardioprotective therapeutic interventions aimed mainly at mitochondria could portend a better protection against myocardial ischemic injury.

## Figures and Tables

**Table 1:** Baseline and post-treatment variables *d*P/dt_max_ and *d*P/dt_min_ at 60-min reperfusion in time control (TC), ischemia reperfusion (IR), cardioplegia (CP), hypothermia (H), and cardioplegia + hypothermia (HCP) groups. *N* = 6 hearts in each group. Values are mean ± SE.  ^∗^*p* < 0.05, compared to TC group;  ^#^*p* < 0.05 compared to IR group;  ^$^*p* < 0.05 compared to IR + CP group.

Time (60-min REP)	TC	IR	IR + CP	IR + H	IR + HCP
*d*P/dt_max_ (mmHg/min)	3549 ± 49	503 ± 93^∗^	1610 ± 45^∗#^	2860 ± 20^∗#$^	3507 ± 73^#$^
*d*P/dt_min_ (mmHg/min)	−2916 ± 130	−359 ± 38^∗^	−1484 ± 76^∗#^	−2206 ± 72^∗#$^	−2799 ± 51^#$^

**Table 2:** Mitochondrial redox state (NADH/FAD) at 25-min ischemia (ISC) and 20-min reperfusion (REP). TC: time control; IR: ischemia and reperfusion without intervention; IR + CP: IR plus cardioplegia treatment; IR + H: IR plus hypothermia treatment; HCP: and hypothermia plus cardioplegia treatment + IR. *N* = 6 hearts in each group. Values represent mean ± SE.  ^∗^*p* < 0.05 compared to TC group;  ^#^*p* < 0.05 compared to IR group;  ^$^*p* < 0.05 compared to IR + CP group.

Time (min)	TC	IR	IR + CP	IR + H	IR + HCP
NADH (AFU)					
25-min ISC	1.30 ± 0.04	1.45 ± 0.03^∗^	1.50 ± 0.03^∗^	1.92 ± 0.07^∗#$^	1.83 ± 0.06^∗#$^
20-min REP	1.30 ± 0.06	1.26 ± 0.04	1.27 ± 0.11	1.41 ± 0.10	1.40 ± 0.11
FAD (AFU)					
25-min ISC	0.24 ± 0.01	0.26 ± 0.04	0.21 ± 0.02	0.21 ± 0.01	0.21 ± 0.02
20-min REP	0.24 ± 0.02	0.24 ± 0.04	0.25 ± 0.03	0.24 ± 0.02	0.23 ± 0.02

## Data Availability

All data supporting the conclusions of this study are provided in the article.

## Supplementary Materials

Supplementary 1 Supplementary Table 1. Baseline and post-treatment parameters of *d*P/*d*t_max_ and *d*P/*d*t_min_ at 5 and 20 min reperfusion in time control (TC), ischemia reperfusion (IR), cardioplegia (CP), hypothermia (H), and hypothermia + cardioplegia (CP + H) groups. Values are mean ± SE.  ^∗^*p* < 0.05 compared to TC group;  ^#^*p* < 0.05 compared to IR group;  ^$^*p* < 0.05 compared to IR + CP group.

Supplementary 2 Supplementary Figure S1. Coronary flow (CF), diastolic LVP (DiaLVP), developed LVP (dLVP), and rate pressure product (RPP) at 5 (a, b, c, and d) and 20 (e, f, g, and h) min reperfusion in time control (TC), ischemia reperfusion (IR), cardioplegia (CP), hypothermia (H), and cardioplegia + hypothermia (CP + H) groups. Values are mean ± SE.  ^∗^*p* < 0.05 compared to TC group;  ^#^*p* < 0.05 compared to IR group.

Supplementary 3 Supplementary Figure S2. Representative traces of O_2_ consumption rates (insets: average respiratory control index (RCI)) at 5 (a) and 20 (b) min reperfusion periods in time control (TC), ischemia reperfusion (IR), cardioplegia (CP), hypothermia (H), and cardioplegia + hypothermia (CP + H) groups. Change in membrane potential (*ΔΨ*_m_) (insets: average time (S3 time (sec) for repolarization after ADP-induced depolarization (state 3)) at 5 (c) and 20(d) min reperfusion periods Mitochondria were energized with Na^+^-Succinate (complex II substrate). The bar graphs show mean ± SE.  ^∗^*p* < 0.05 compared to TC group;  ^#^*p* < 0.05 compared to IR.

Supplementary 4 Supplementary Figure S3. Representative traces of mitochondrial calcium retention capacity (CRC) at 5 (a) and 20 (b) min reperfusion periods in time control (TC), ischemia reperfusion (IR), cardioplegia (CP), hypothermia (H), and cardioplegia + hypothermia (CP + H) groups. The insets are representative traces at later time points that show in greater details the differences in the kinetics of mitochondrial Ca^2+^ uptake during the CaCl_2_ pulse challenges until mitochondria stopped taking Ca^2+^. CCCP, the mitochondrial uncoupler, was given after mitochondria stopped taking the added Ca^2+^ to unload all the Ca^2+^ sequestered during bolus additions. Mitochondria were energized with Na^+^-Succinate (complex II substrate).

Supplementary 5 Supplementary Figure S4. Western blot analysis of Bax at 5 (a), 20 (b), and 60 (c) min reperfusion periods in time control (TC), ischemia reperfusion (IR), cardioplegia (CP), hypothermia (H), and cardioplegia + hypothermia (CP + H) groups. *N*  = mitochondria from 3 to 4 hearts in each group. There was no significant difference in Bax level in cytosolic and mitochondrial fractions between the groups at any reperfusion time.
