# Supplementary material for: Hypothermia Prevents Cardiac Dysfunction during Acute Ischemia Reperfusion by Maintaining Mitochondrial Bioenergetics and by Promoting Hexokinase II Binding to Mitochondria
Source: Oxid Med Cell Longev. 2022 Jul 13;2022:4476448. doi: 10.1155/2022/4476448 (PMC9301761; doi:10.1155/2022/4476448)
Supplement: Supplementary 1 — Supplementary Table 1. Baseline and post-treatment parameters of dP/dtmax and dP/dtmin at 5 and 20 min reperfusion in time control (TC), ischemia reperfusion (IR), cardioplegia (CP), hypothermia (H), and hypothermia + cardioplegia (CP + H) groups. Values are mean ± SE. ∗p < 0.05 compared to TC group; #p < 0.05 compared to IR group; $p < 0.05 compared to IR + CP group. [file 4476448.f1.pdf]

Time (min REP)	TC	IR	IR+CP	IR+H	IR+HCP
*d*P/dt_{max} (mmHg/min)					
5	3420 ± 111	379 ± 156*	1524 ± 46$^{*\#}$	2748 ± 105$^{*\#\$}$	3320 ± 157$^{\#\$}$
20	3748 ± 110	356 ± 79*	1778 ± 118$^{*\#}$	2950 ± 84$^{*\#\$}$	3595 ± 185$^{\#\$}$
*d*P/dt_{min} (mmHg/min)					
5	-3250 ± 84	-340 ± 131*	-1290 ± 94$^{*\#}$	-1781 ± 108$^{*\#}$	-3200 ± 157$^{\#}$
20	-3145 ± 120	-350 ± 75*	-1300 ± 66$^{*\#}$	-1893 ± 108$^{*\#}$	-3022 ± 58$^{\#}$
