# Supplementary figures and images for: Hypothermia Prevents Cardiac Dysfunction during Acute Ischemia Reperfusion by Maintaining Mitochondrial Bioenergetics and by Promoting Hexokinase II Binding to Mitochondria
Source: Oxid Med Cell Longev. 2022 Jul 13;2022:4476448. doi: 10.1155/2022/4476448 (PMC9301761; doi:10.1155/2022/4476448)

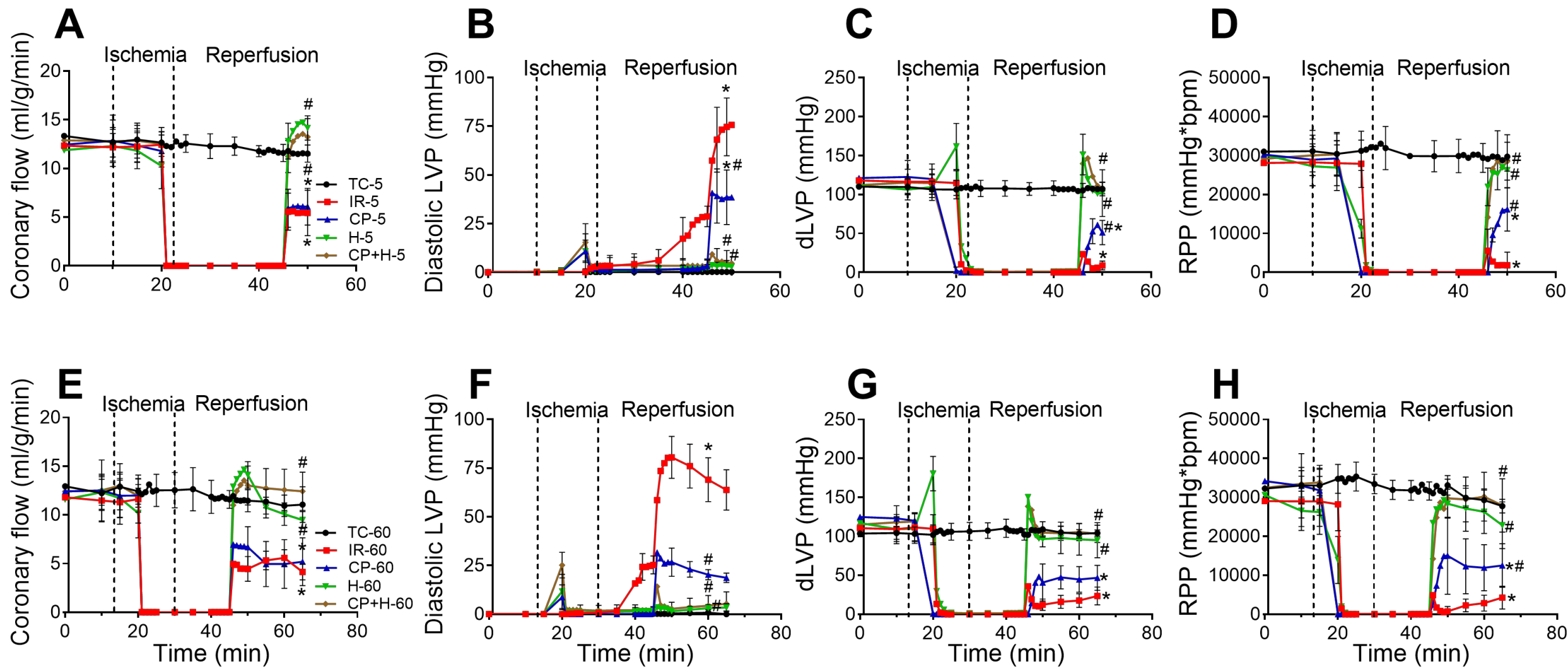

Supplement: Supplementary 2 — Supplementary Figure S1. Coronary flow (CF), diastolic LVP (DiaLVP), developed LVP (dLVP), and rate pressure product (RPP) at 5 (a, b, c, and d) and 20 (e, f, g, and h) min reperfusion in time control (TC), ischemia reperfusion (IR), cardioplegia (CP), hypothermia (H), and cardioplegia + hypothermia (CP + H) groups. Values are mean ± SE. ∗p < 0.05 compared to TC group; #p < 0.05 compared to IR group. [file 4476448.f2.pdf]

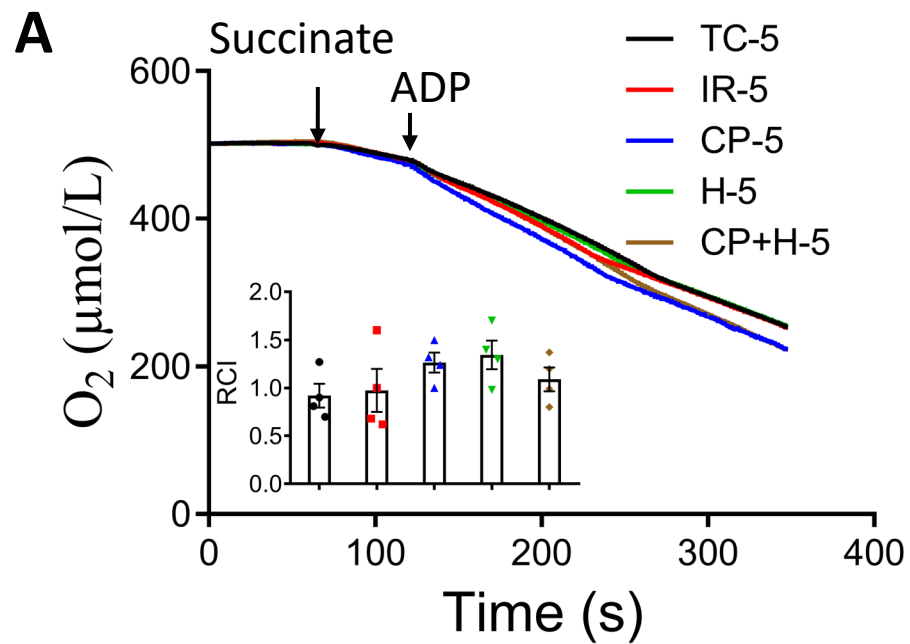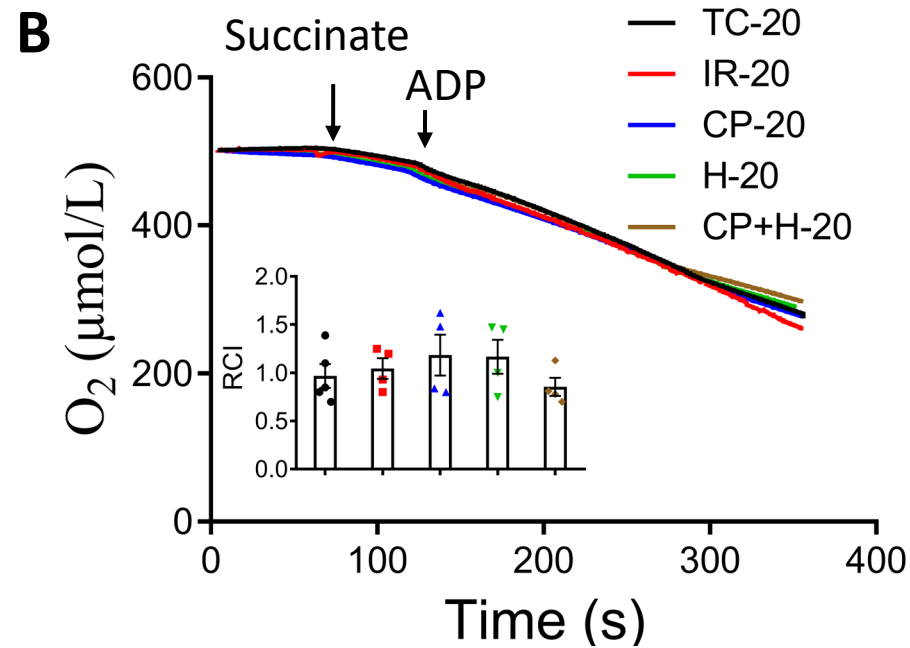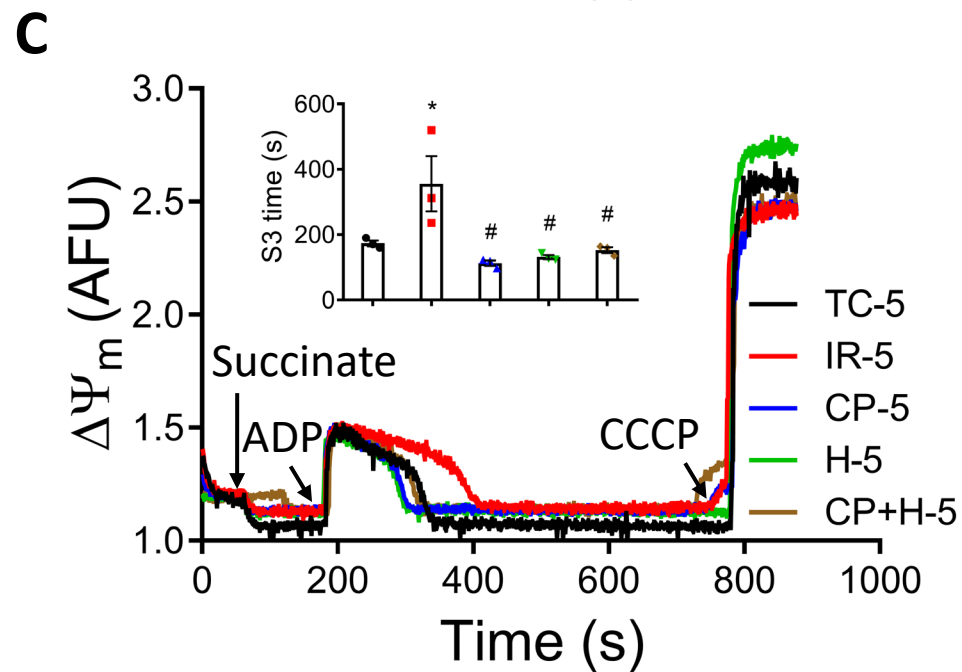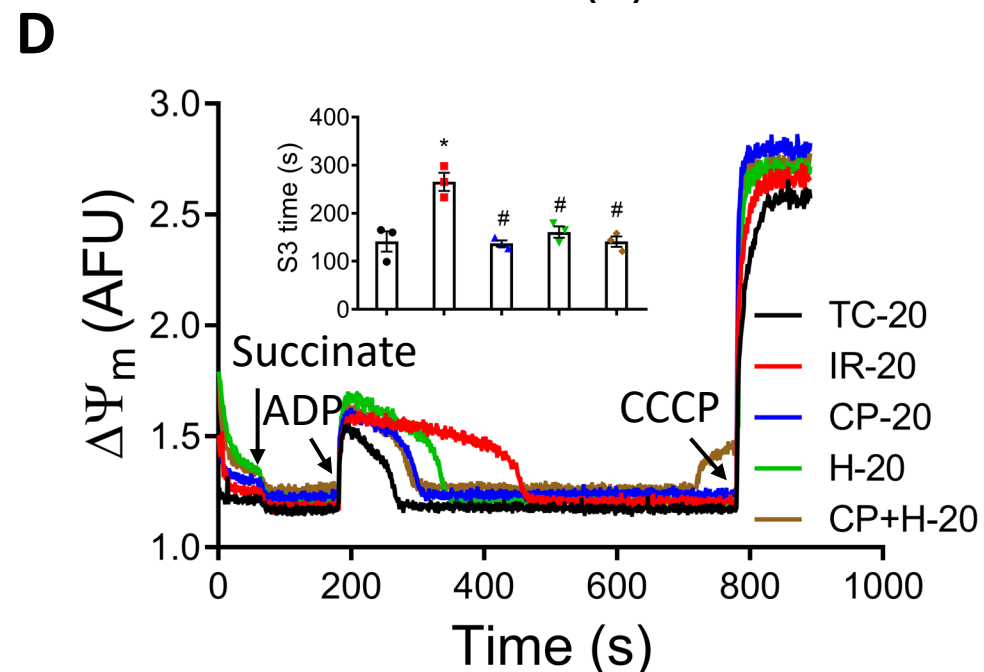

Supplement: Supplementary 3 — Supplementary Figure S2. Representative traces of O2 consumption rates (insets: average respiratory control index (RCI)) at 5 (a) and 20 (b) min reperfusion periods in time control (TC), ischemia reperfusion (IR), cardioplegia (CP), hypothermia (H), and cardioplegia + hypothermia (CP + H) groups. Change in membrane potential (ΔΨm) (insets: average time (S3 time (sec) for repolarization after ADP-induced depolarization (state 3)) at 5 (c) and 20(d) min reperfusion periods Mitochondria were energized with Na+-Succinate (complex II substrate). The bar graphs show mean ± SE. ∗p < 0.05 compared to TC group; #p < 0.05 compared to IR. [file 4476448.f3.pdf]

**A**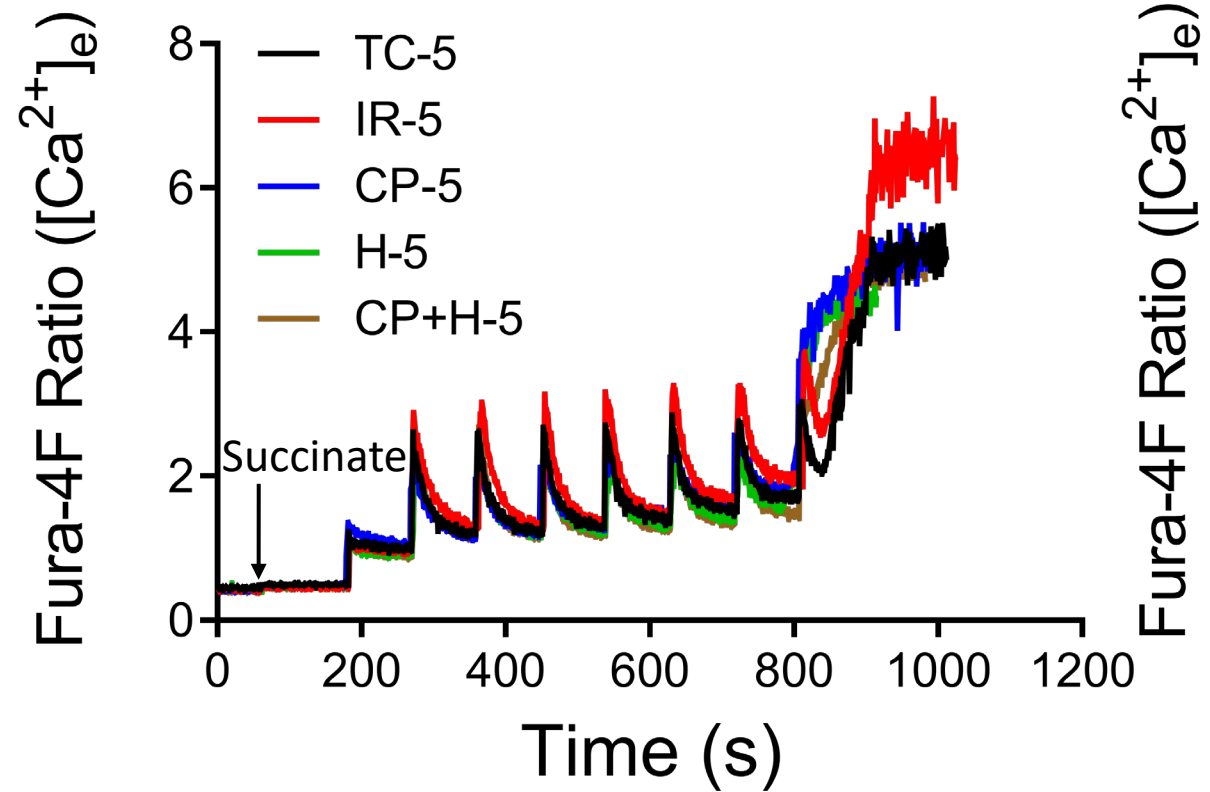**B**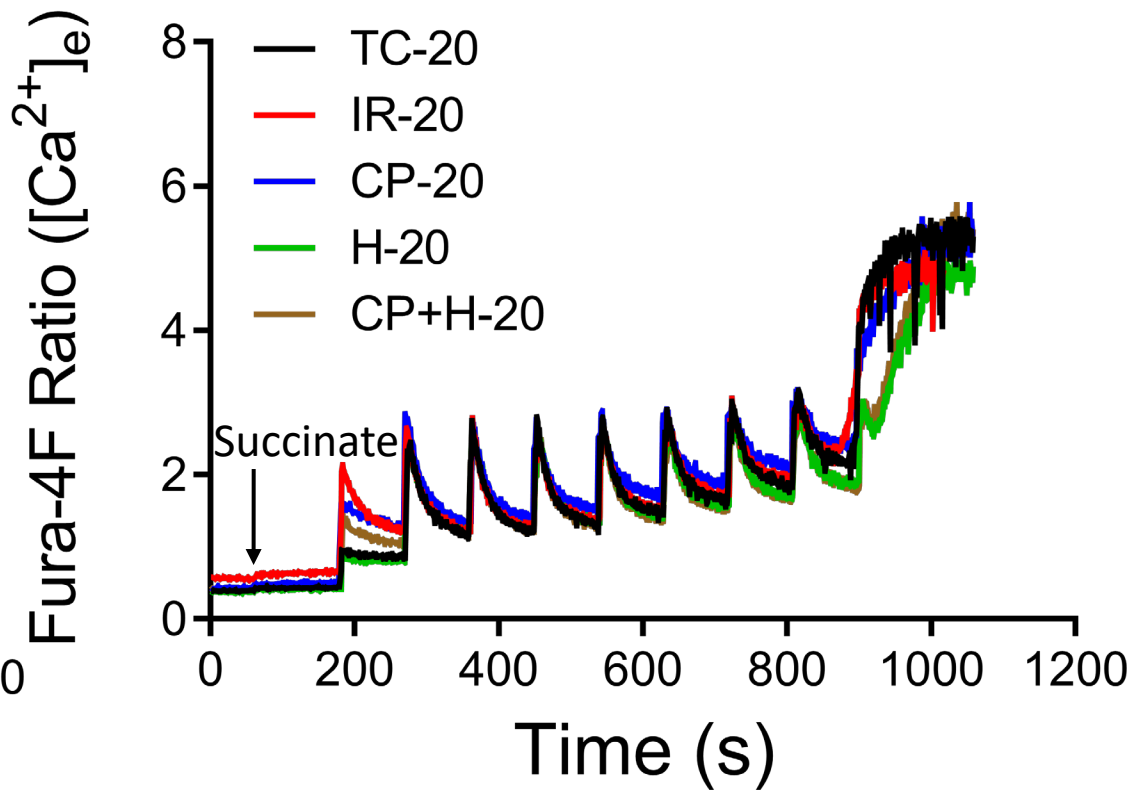

Supplement: Supplementary 4 — Supplementary Figure S3. Representative traces of mitochondrial calcium retention capacity (CRC) at 5 (a) and 20 (b) min reperfusion periods in time control (TC), ischemia reperfusion (IR), cardioplegia (CP), hypothermia (H), and cardioplegia + hypothermia (CP + H) groups. The insets are representative traces at later time points that show in greater details the differences in the kinetics of mitochondrial Ca2+ uptake during the CaCl2 pulse challenges until mitochondria stopped taking Ca2+. CCCP, the mitochondrial uncoupler, was given after mitochondria stopped taking the added Ca2+ to unload all the Ca2+ sequestered during bolus additions. Mitochondria were energized with Na+-Succinate (complex II substrate). [file 4476448.f4.pdf]

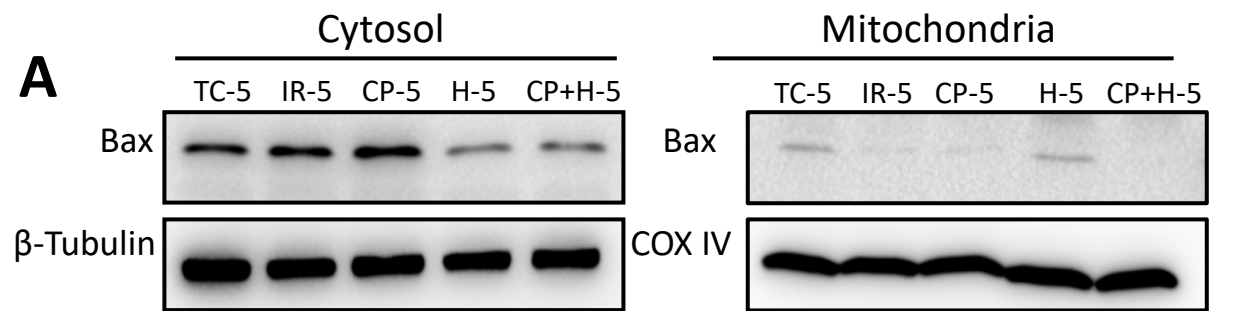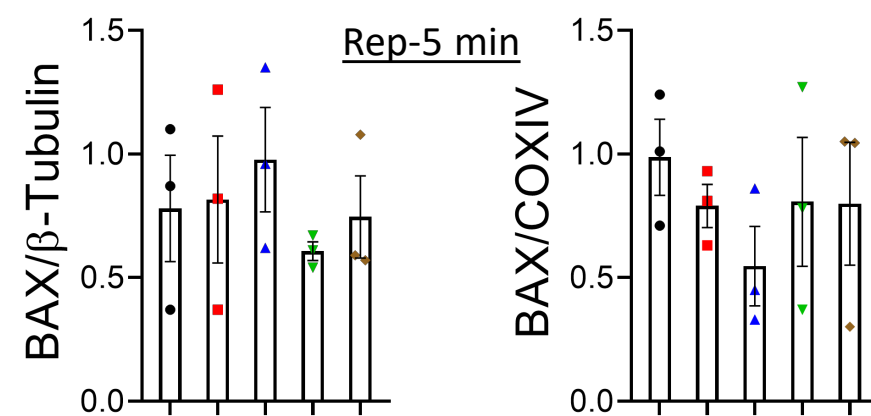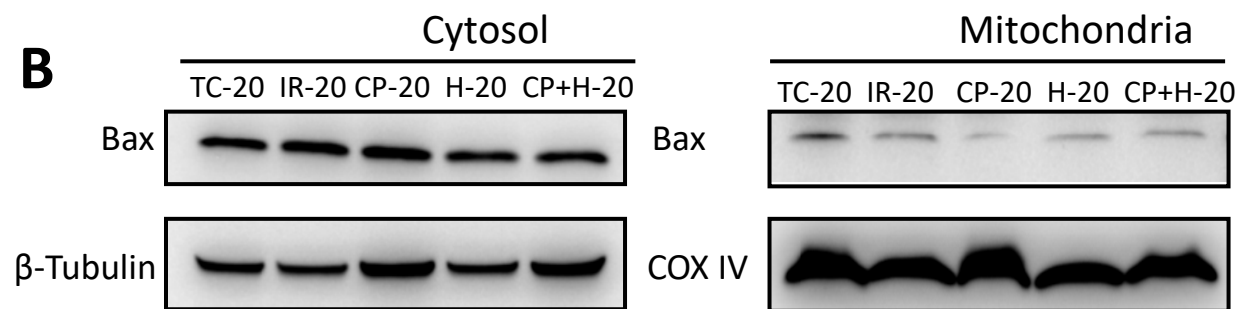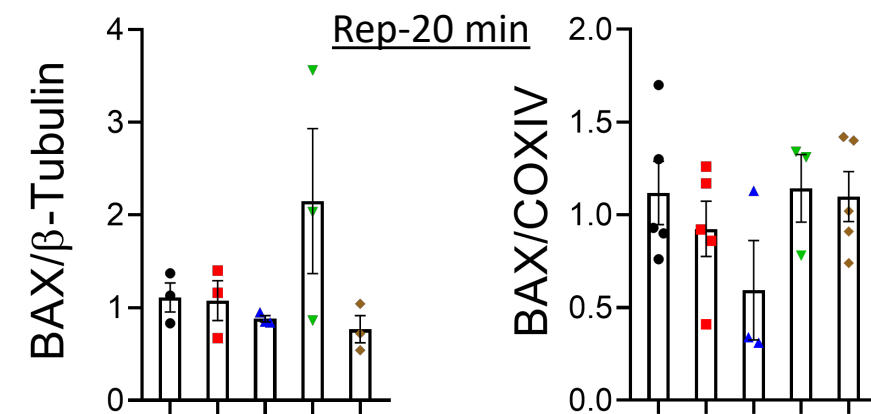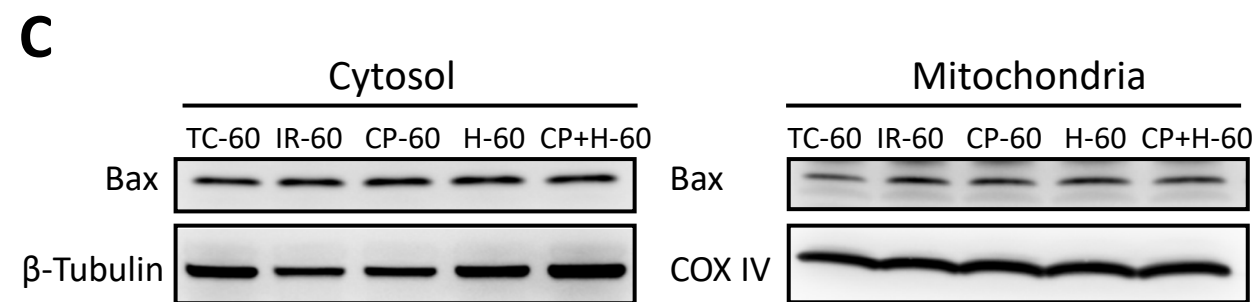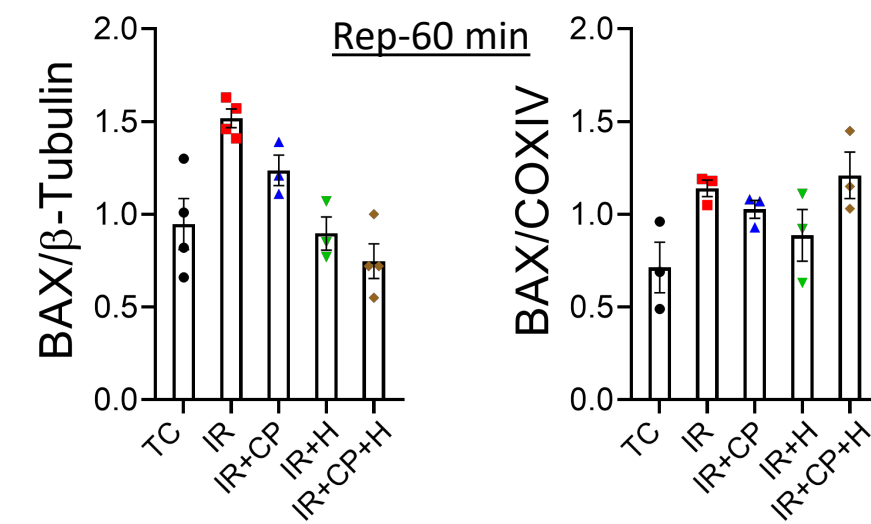

Supplement: Supplementary 5 — Supplementary Figure S4. Western blot analysis of Bax at 5 (a), 20 (b), and 60 (c) min reperfusion periods in time control (TC), ischemia reperfusion (IR), cardioplegia (CP), hypothermia (H), and cardioplegia + hypothermia (CP + H) groups. N = mitochondria from 3 to 4 hearts in each group. There was no significant difference in Bax level in cytosolic and mitochondrial fractions between the groups at any reperfusion time. [file 4476448.f5.pdf]
